# The AP-2 Transcription Factor APTF-2 Is Required for Neuroblast and Epidermal Morphogenesis in *Caenorhabditis elegans* Embryogenesis

**DOI:** 10.1371/journal.pgen.1006048

**Published:** 2016-05-13

**Authors:** Yemima Budirahardja, Pei Yi Tan, Thang Doan, Peter Weisdepp, Ronen Zaidel-Bar

**Affiliations:** 1 Mechanobiology Institute, National University of Singapore, Singapore; 2 Department of Genome Sciences, University of Washington, Seattle, Washington, United States of America; 3 Department of Biomedical Engineering, National University of Singapore, Singapore; University of California San Diego, UNITED STATES

## Abstract

The evolutionarily conserved family of AP-2 transcription factors (TF) regulates proliferation, differentiation, and apoptosis. Mutations in human AP-2 TF have been linked with bronchio-occular-facial syndrome and Char Syndrome, congenital birth defects characterized by craniofacial deformities and patent ductus arteriosus, respectively. How mutations in AP-2 TF cause the disease phenotypes is not well understood. Here, we characterize the *aptf-2(qm27)* allele in *Caenorhabditis elegans*, which carries a point mutation in the conserved DNA binding region of AP-2 TF. We show that compromised APTF-2 activity leads to defects in dorsal intercalation, aberrant ventral enclosure and elongation defects, ultimately culminating in the formation of morphologically deformed larvae or complete arrest during epidermal morphogenesis. Using cell lineaging, we demonstrate that APTF-2 regulates the timing of cell division, primarily in ABarp, D and C cell lineages to control the number of neuroblasts, muscle and epidermal cells. Live imaging revealed nuclear enrichment of APTF-2 in lineages affected by the *qm27* mutation preceding the relevant morphogenetic events. Finally, we found that another AP-2 TF, APTF-4, is also essential for epidermal morphogenesis, in a similar yet independent manner. Thus, our study provides novel insight on the cellular-level functions of an AP-2 transcription factor in development.

## Author Summary

Mutations in the evolutionarily conserved family of AP-2 transcription factors are associated with multiple birth defects in Char syndrome and Brancio-oculo-facial syndrome. These DNA-binding proteins are known to regulate the proliferation, differentiation and death of specific cells during embryonic development but how point mutations in the AP-2 DNA-binding domain lead to these diseases during development is currently unknown. We have identified a mutation in one of the AP-2 orthologs of the nematode *Caenorhabditis elegans*, APTF-2, which falls in the same mutation hotspot as in human Char syndrome and Brancio-oculo-facial syndrome patients. Compromised APTF-2 activity in *C*. *elegans* results in embryonic lethality and embryos that survive to hatching displays body morphological defects, reminiscent of the aforementioned human diseases. Using time-lapse microscopy, we found that misregulation of cell division in the skin, muscle and neuronal cell lineages is the primary cause of developmental arrest. Our study provides insight into the regulation of cell division timing by AP-2 transcription factors and provides a model to study human diseases associated with AP-2 mutations.

## Introduction

The AP-2 family of transcription factors is associated with proper development of mammals by maintaining a balance between cell proliferation and cell death [1, 2]. Five members of the AP-2 family have been identified in vertebrates: AP-2α, AP-2β, AP-2γ AP-2δ and AP-2ε [3–8]. All AP-2 transcription factors have a central basic region followed by a highly conserved helix-span-helix (HSH) motif at the carboxyl terminus [9]. The HSH is essential for dimerization and together with the adjacent basic region achieves a sequence-specific DNA binding function [10]. The less conserved proline- and glutamine-rich region at the amino terminus is required for transcription activation [11]. AP-2 transcription factors bind primarily to the palindromic core sequence 5’-GCCN_3_GGC-3’ and serve a dual role as transcriptional activators or repressors [1].

AP-2 knockout mice display a wide spectrum of anomalies in early development such as craniofacial, neural tube and body wall defects, and polycystic kidney disease associated with uncontrolled apoptosis [12–14]. The phenotypic defects correspond to the diverse and overlapping expression patterns of murine AP-2 family genes in the neural crest cells, forebrain, facial and limb mesenchyme, and various types of epithelial cells [4, 5, 15, 16]. In humans, mutations in TF AP-2-alpha (TFAP2A) have been associated with branchio-oculo-facial syndrome (BOFS), a congenital birth defect characterized by craniofacial abnormalities, skin and eye defects as well as hearing problems [17]. Char Syndrome, a congenital disease characterized by patent ductus arteriosus and facial and hand anomalies, was linked to mutations in TF AP-2-beta (TFAP2B) [18]. Multiple point mutations and deletions in BOFS and Char Syndrome patients have been mapped to the conserved basic region of the DNA binding domain in AP-2α and AP-2β [17–20]. However, the molecular mechanisms by which these mutations manifest in the disease symptoms are not well understood.

In *C*. *elegans*, there are four AP-2 TF family members: APTF-1, APTF-2, APTF-3 and APTF-4. APTF-1 functions in the GABAergic neuron RIS to induce sleep-like quiescence in *C*. *elegans* [21]. Other APTF members have not yet been studied. Using whole genome sequencing, we identified a mutant allele which gave rise to a single amino acid change in the basic region of APTF-2. Here, we describe the role of APTF-2 during *C*. *elegans* embryonic development, specifically during epidermal morphogenesis that involves the formation of a single epithelial layer that envelops the animal. We found APTF-2 is important for epithelial dorsal intercalation and ventral enclosure and mutation of *aptf-2* results in larva with body morphology defects as well as embryonic lethality. Cell lineaging revealed misregulation of cell division timing, possibly leading to the phenotypic defects. Thus, *C*. *elegans* could serve as a model to study molecular and cellular consequences of mutations in the family of AP-2 TF analogous to those mutations in human AP-2 TF underlying BOFS and Char syndrome diseases.

## Results

### A missense mutation in *aptf-2*(*qm27*) causes embryonic lethality and morphological defects in larva

**Fig 1:**
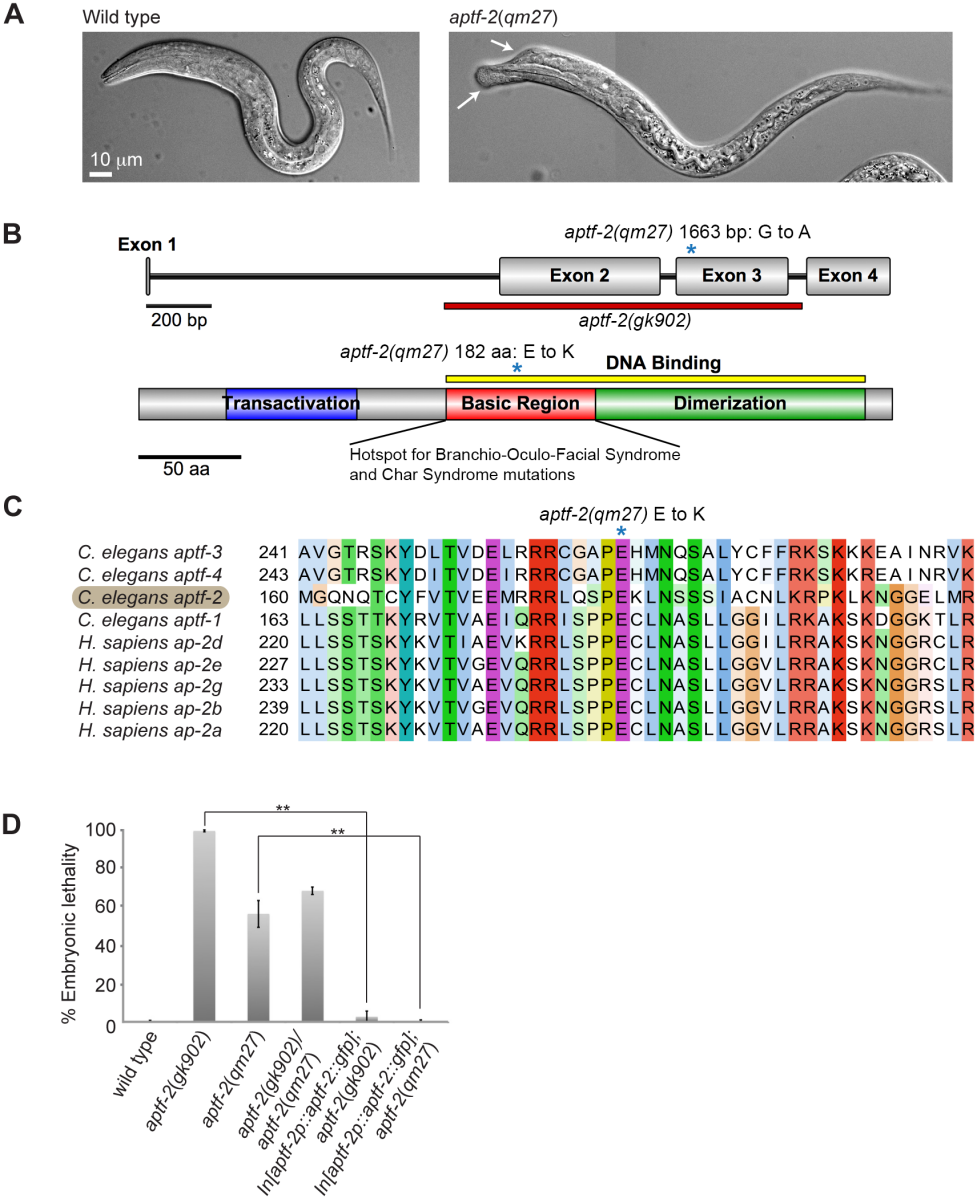
A missense mutation in *aptf-2*(*qm27*) gives rise to embryonic lethality and morphologically defective larva. (A) Wild-type L1 larva (left panel) and *aptf-2*(*qm27*) (right panel) L1 larva exhibiting morphological defects in the head (indicated by the arrows). (B) Gene and protein organization of APTF-2. *qm27* is an *aptf-2* allele with a missense mutation at amino acid 182 (indicated by an asterisk) in the basic region of the DNA binding domain converting glutamic acid to lysine, whereas *gk902* is a frame shift deletion allele of *aptf-2* spanning from the middle of intron 1 to the end of exon 3. (C) Multiple alignment of AP-2 transcription factors in *C*. *elegans* and *H*. *sapiens* shows that the glutamic acid residue mutated in *qm27* is conserved across species. (D) Loss of *aptf-2* function in *aptf-2*(*gk902*), *aptf-2*(*qm27*) and in trans heterozygote *aptf-2*(*gk902*)/*aptf-2*(*qm27*) animals results in high embryonic lethality. Stable expression of APTF-2::GFP rescues embryonic lethality in *aptf-2*(*qm27*) and *aptf-2*(*gk902*) worms. Data are presented as mean ± s.e.m. n > 500 embryos from five independent experiments (*P<0.05, **P<0.01, two-tailed test). See S1 Movie for rescue experiment and S2 Table for the numerical values and statistical analysis.

In a genetic screen for maternal-effect mutations that have an impact on *C*. *elegans* development Hekimi *et*. *al*. isolated *mal-1(qm27)* as a mutation that causes extensive embryonic and larval lethality, with surviving homozygous mutants displaying morphological defects characterized by dorsal protrusions on the head and/or shortened body length [22] (Figs 1A and S1A and Tables 1 and S1). Genetic mapping predicted the approximate location of *mal-1(qm27)* on chromosome IV [22], but the molecular identity of the *mal-1* gene has remained unknown. Whole genome sequencing of a *mal-1(qm27)* strain identified a missense mutation in *aptf-2*, one of four AP-2-like transcription factors in *C*. *elegans*. This mutation changes a highly conserved glutamic acid residue within the basic region of the DNA binding domain into a lysine residue (Fig 1B and 1C). Previous findings have indicated the basic region as a mutation hotspot for BOFS and Char Syndrome [17–20]. We also analysed *gk902*, a deletion allele of *aptf-2* generated by the International *C*. *elegans* Gene Knockout Consortium. Similar to *qm27*, *gk902* worms also displayed maternal effect embryonic lethality with 99 ± 0.5% of embryos not hatching and the few hatching larva displaying head and/or tail morphological defects and arresting as larva (Table 1, S1 Table). The *gk902* and *qm27* alleles failed to complement each other, as the progeny of trans-heterozygote *aptf-2*(*gk902*)/*aptf-2*(*qm27*) had a level of embryonic lethality in between homozygote *aptf-2(qm27)* and homozygote *aptf-2(gk902)*, consistent with them being mutations in the same gene (Fig 1D and S2 Table). Moreover, expression of APTF-2::GFP from an integrated array driven by the *aptf-2* promoter, completely rescued the embryonic lethality in both *aptf-2(gk902)* and *aptf-2(qm27)* strains (Fig 1D, S2 Table, S1 Movie), confirming the embryonic lethality in these strains is due to the mutations in *aptf-2*. Consistent with Hekimi *et*. *al*. [22] we found that *qm27* homozygous progeny of *+/qm27* worms are phenotypically normal, indicating maternal rescue.

**Table 1:** Brood size, percentage of embryonic lethality and percentage of larval arrest of *aptf-2* mutant worms.

Genotypes	n	Embryos laid/ hermaphrodite	% Dead embryos	% Larval arrest
N2	10	320 ± 20	0.3 ± 0.4	0.3 ± 0.4
*aptf-2*(*gk902*)	9	291 ± 40	99 ± 0.5	100
*aptf-2*(*qm27*)	9	277 ± 18	56 ± 7	92 ± 2

Mean number of embryos laid/ hermaphrodite, % dead embryos and % larval arrest ± s.e.m. are indicated. Note: We scored F2 homozygous *gk902* because the F1 had maternal contribution from their heterozygous mothers.

### *aptf-2(qm27)* embryos fail during epidermal morphogenesis

To characterize the developmental defects in *aptf-2(qm27)* embryos leading to their lethality we used 4D differential interference contrast (DIC) microscopy to follow isolated embryos positioned with either their dorsal or ventral side facing the microscope objective. We identified three major defects, all related to epidermal morphogenesis: failure in dorsal epidermal cell intercalation, failure of ventral epidermal cell enclosure, and arrest during elongation (Table 2). A small percentage of embryos also exhibited leakage of cells out of the body of the embryo during elongation (Table 2). The exact cause for elongation arrest is not easily discerned, but we noted that one third of the ventrally-oriented embryos that arrested during elongation had previous ventral enclosure defects and nearly all of the dorsally-oriented embryos that arrested in elongation displayed earlier defects in dorsal intercalation.

**Table 2:** Phenotypic analysis of *aptf-2*(*qm27*) embryos analyzed by DIC.

Embryo orientation	n	% Embryonic phenotypes				
		Wild- type	Dorsal intercalation defect	Ventral enclosure defect	Elongation arrest	Cell leakage during elongation
Dorsal	30	28	64	n.a.	68	4
Ventral	51	12	n.a.	31	82	6

**Fig 2:**
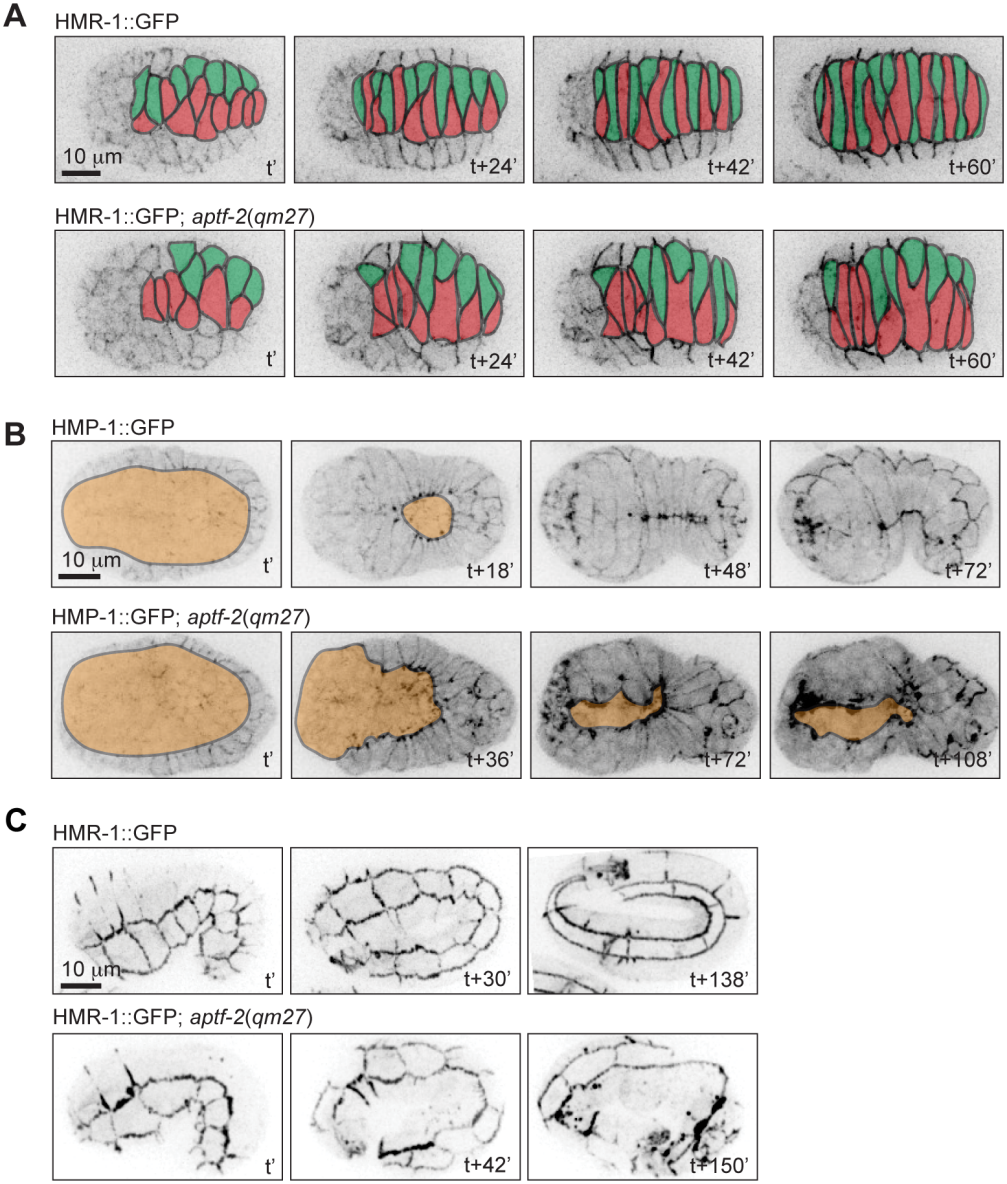
*aptf-2*(*qm27*) embryos are defective in epidermal morphogenesis. (A) Dorsal intercalation in a wild-type embryo (top panels) and failed intercalation in an *aptf-2*(*qm27*) embryo (bottom panels). Cell junctions are visualized by E-cadherin/HMR-1::GFP and cells from opposite rows are colored to better appreciate the intercalation process. t’ marks the time when two rows of dorsal epidermal cells are aligned and ready to undergo intercalation. t+60’ marks the end of the intercalation process in wild-type embryo. See corresponding S2 Movie. (B) Ventral enclosure in a wild-type embryo (top panels) proceeds until cells from opposite sides meet at the ventral midline, whereas in the *aptf-2*(*qm27*) embryo (bottom panels), the ventral epidermis fails to enclose the embryo. Cell junctions are visualized by alpha-catenin/HMP-1::GFP and the cells underlying the ventral epidermis are artificially colored in orange to highlight the enclosure process. t’ marks the beginning of ventral enclosure, while t+48’ and t+72’ in wild type embryo mark the completion of ventral enclosure and the beginning of elongation process, respectively. See corresponding S3 Movie. (C) In a little over 2h the wild-type embryo (top panels) elongates from the comma stage to over 3fold the eggshell length, whereas the *aptf-2*(*qm27*) embryo (bottom panels) arrests at approximately the twofold length. t’ marks the beginning of elongation, whereas t+138’ marks 3 fold elongation in wild type. Cell junctions are visualized by E-cadherin/HMR-1::GFP. In this and the subsequent figures, all embryos are oriented with anterior on the left and dorsal on the top.

We confirmed the phenotypes observed in DIC microscopy by imaging *aptf-2(qm27)* embryos expressing fluorescently-tagged cell-cell junction markers E-cadherin/HMR-1 and alpha-catenin/HMP-1. As shown in Fig 2, S2 and S3 Movies, these markers confirmed the failure of epidermal cells to dorsally intercalate (Fig 2A), ventrally migrate (Fig 2B), and elongate the embryo (Fig 2C). Previous studies have shown that ventral enclosure defects are often preceded by failure of ventral neuroblasts to seal the cleft at the end of gastrulation. We imaged gastrulation cleft closure in wild-type and *aptf-2(qm27)* embryos by DIC and by expression of the neuroblast marker KAL-1::GFP and found that the ventral cleft in the mutant embryos was larger to begin with, took up to four times the amount of time to close and in some cases did not completely close before the onset of epidermal ventral enclosure (S1 Fig).

### *aptf-2(gk902)* embryos display excessive apoptosis in the early embryo in addition to epidermal morphogenesis defects

**Fig 3:**
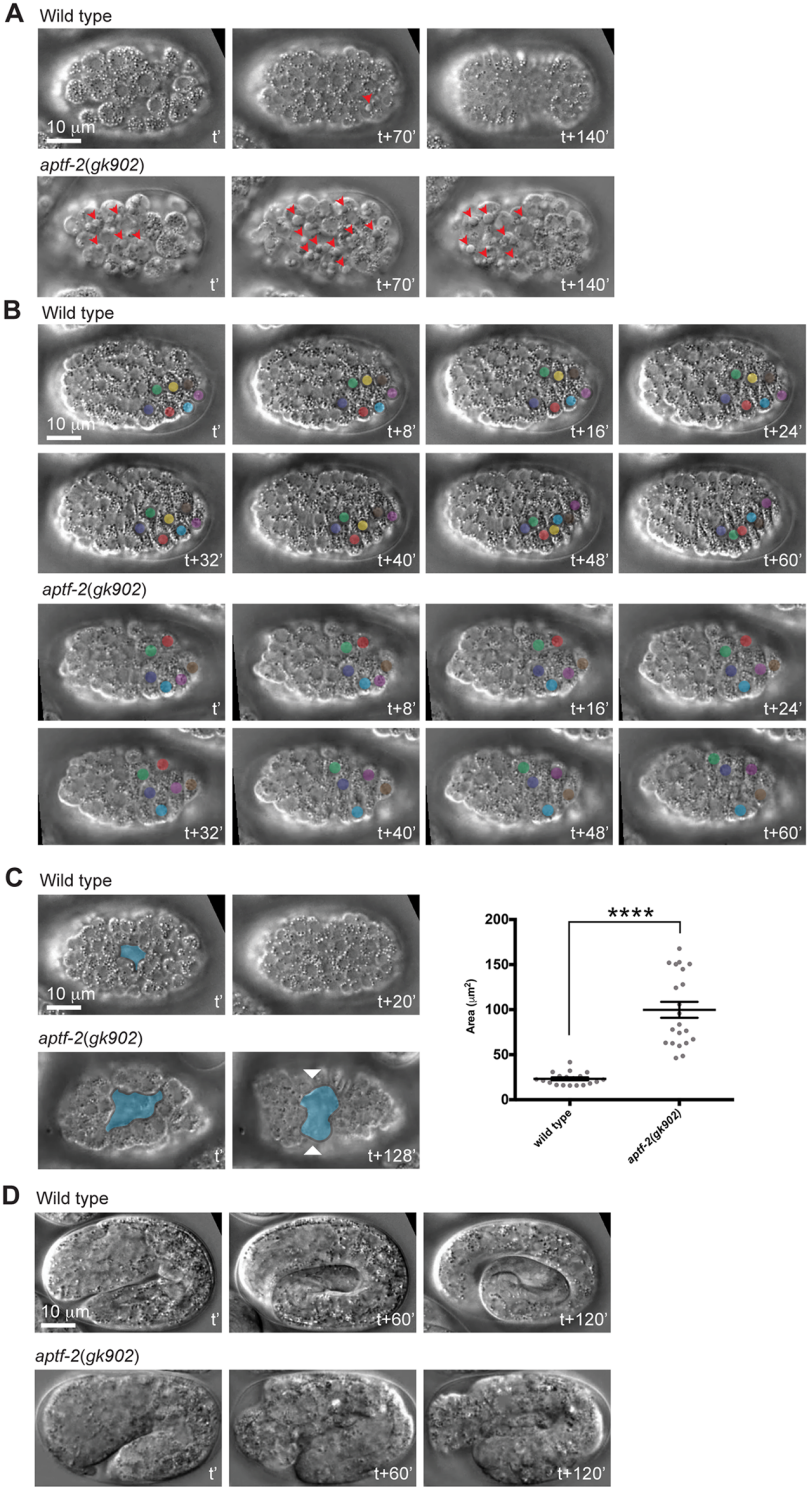
Complete loss-of-function of *aptf-2* results in excessive apoptosis and epidermal morphogenesis defects during embryogenesis. (A) While only few cells in the wild-type embryo undergo apoptosis (top panels), substantial number of cells undergo apoptosis in the *aptf-2*(*gk902*) embryo (bottom panels). Red arrowheads denote apoptotic cells. t’ marks mid gastrulation, while t+140’ marks ventral enclosure in wild type. (B) In the wild type embryo, two rows of dorsal epidermal cells intercalate to form one row of cells during the first stage of the epidermal morphogenesis. This process is accompanied by movement of the dorsal epidermal nuclei towards the opposite side of their starting position (top panels). Intercalation of dorsal epidermal cells is defective in *aptf-2*(*gk902*) embryo as indicated by abnormal or absence of movement of dorsal epidermal nuclei (bottom panels). Nuclei of dorsal epidermal cells are colored to better visualize their positions during the intercalation process. t’ marks the positioning of the two rows of epidermal cells at the dorsal side, whereas t+60’ in wild type marks the completion of the intercalation process. (C) The end of gastrulation is marked by the closure of the ventral cleft. In wild-type embryos, the size of the ventral cleft was 23.14 μm^2^ ± 1.595 (mean ± S.E.M, N = 19) and it closed within 20 minutes (top panels), whereas in the *aptf-2*(*gk902*) embryos, the ventral cleft was four times as large at 99.74 μm^2^ ± 8.858 (mean ± S.E.M, N = 21) and often stayed open until the time of ventral epidermal enclosure and thereby prevented proper epidermal enclosure from taking place (bottom panels). The ventral clefts are colored in blue. t’ marks the start of the ventral cleft and t+20’ marks its closure in wild type. (D) The ventral epidermal enclosure is followed by elongation. While the wild-type embryo progressively elongates from 2- to 3.5-fold in 2 hours (top panels), the *aptf-2*(*gk902*) embryo is arrested during elongation slightly beyond 2 fold stage (bottom panels). t’ marks the beginning of two fold elongation, whereas t+120’ marks 3.5 fold elongation in wild type.

We next examined the embryonic phenotypes of the null mutant *aptf-2(gk902)* by DIC microscopy. We found 60% of the embryos died prior to epidermal morphogenesis, and approximately half of these early embryonic deaths were associated with the appearance of many ectopic apoptotic cells (Fig 3A and Table 3). The remaining 40% of embryos that made it to epidermal morphogenesis all exhibited defects in dorsal intercalation, a quarter of them had ventral enclosure defects, and they all arrested during elongation (Fig 3B, 3C and 3D and Table 3). The massive apoptosis phenotype was completely rescued by the expression of APTF-2::GFP (S2 Table), suggesting that it is a result of the complete loss of APTF-2 function. However, this phenotype was never observed in the partial loss of function allele *qm27*. Neither was it observed in *aptf-2(RNAi)* nor following injection of *aptf-2* dsRNA into *aptf-2(qm27)*.

**Table 3:** Phenotypic analysis of *aptf-2*(*gk902*) embryos analyzed by DIC.

Embryo stage/ orientation	n	% Embryonic phenotypes					
		Wild -type	Early phenotypes		Epidermal morphogenesis phenotypes		
			Apoptosis	Developmental arrest	Dorsal intercalation defect	Ventral enclosure defect	Elongation arrest
Early embryo	87	0	51	49	n.a.	n.a.	n.a.
Epidermal morphogenesis (dorsal)	30	0	n.a.	n.a.	100	n.a.	100
Epidermal morphogenesis (ventral)	27	0	n.a.	n.a.	n.a.	26	100

### *die-1*, a putative APTF-2 target gene is downregulated in *aptf-2(qm27)* embryos

**Fig 4:**
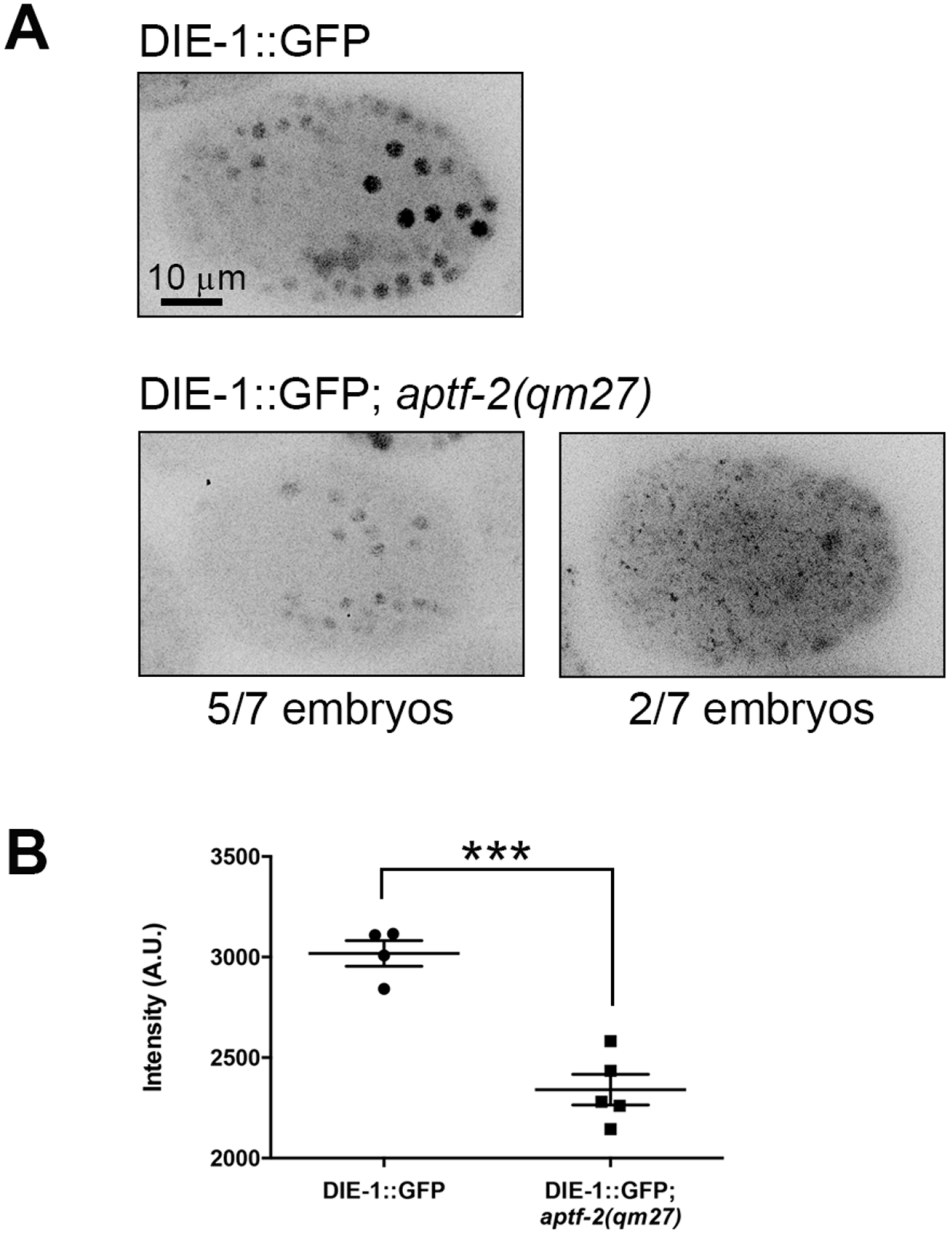
DIE-1 nuclear expression is significantly reduced in *aptf-2 (qm27)* embryos. (A) Nuclear localization pattern of DIE-1::GFP in wild type and *aptf-2 (qm27)* mutant embryos. Five of seven mutant embryos showed wild type-like nuclear localization while two did not have observable nuclear enhancement of DIE-1::GFP. (B) Quantification of the mean intensity of wild type and *aptf-2 (qm27)* mutant embryos showing nuclear localization of DIE-1::GFP. Error bar denotes mean ± sem, p ≤ 0.001.

Using TargetOrtho [23], a phylogenetic footprinting tool to identify transcription factor targets, we identified within the *C*. *elegans* genome 1631 putative AP-2 TF binding sites in the 3KB upstream promoter region of 872 genes (S1 Text). Protein domain analysis of these genes revealed enrichment in F-box, Homeobox, EF-hand, SET and CUB domain proteins, as well as others, and gene onthology analysis of biological processes showed enrichment in genes associated with embryonic development, tissue morphogenesis, locomotion, regulation of growth rate, and reproduction, among others (see S1 Text for full list). Among the putative AP-2 TF regulated genes classified as associated with epithelium development our attention was caught by *die-1*. The zinc finger transcription regulator DIE-1 is autonomously required in the posterior dorsal hypodermis for intercalation, for morphogenesis in other embryonic tissues, and for normal postembryonic growth and vulval development [24, 25]. Given the defects we observed in epidermal morphogenesis we tested whether the expression of *die-1* is altered in *aptf-2* mutants. Indeed, we found that two out of seven *aptf-2(qm27)* embryos showed aberrant localization of DIE-1::GFP. Furthermore, we measured a 22.5% reduction in mean intensity of DIE-1::GFP in the nucleus of mutant embryos with proper nuclear localization (2340 a.u. ± 75.93, n = 5) compared to wild type (3018 a.u. ± 63.68, n = 4) (Fig 4).

### *aptf-2(qm27)* embryo development is retarded compared to wild-type

**Fig 5:**
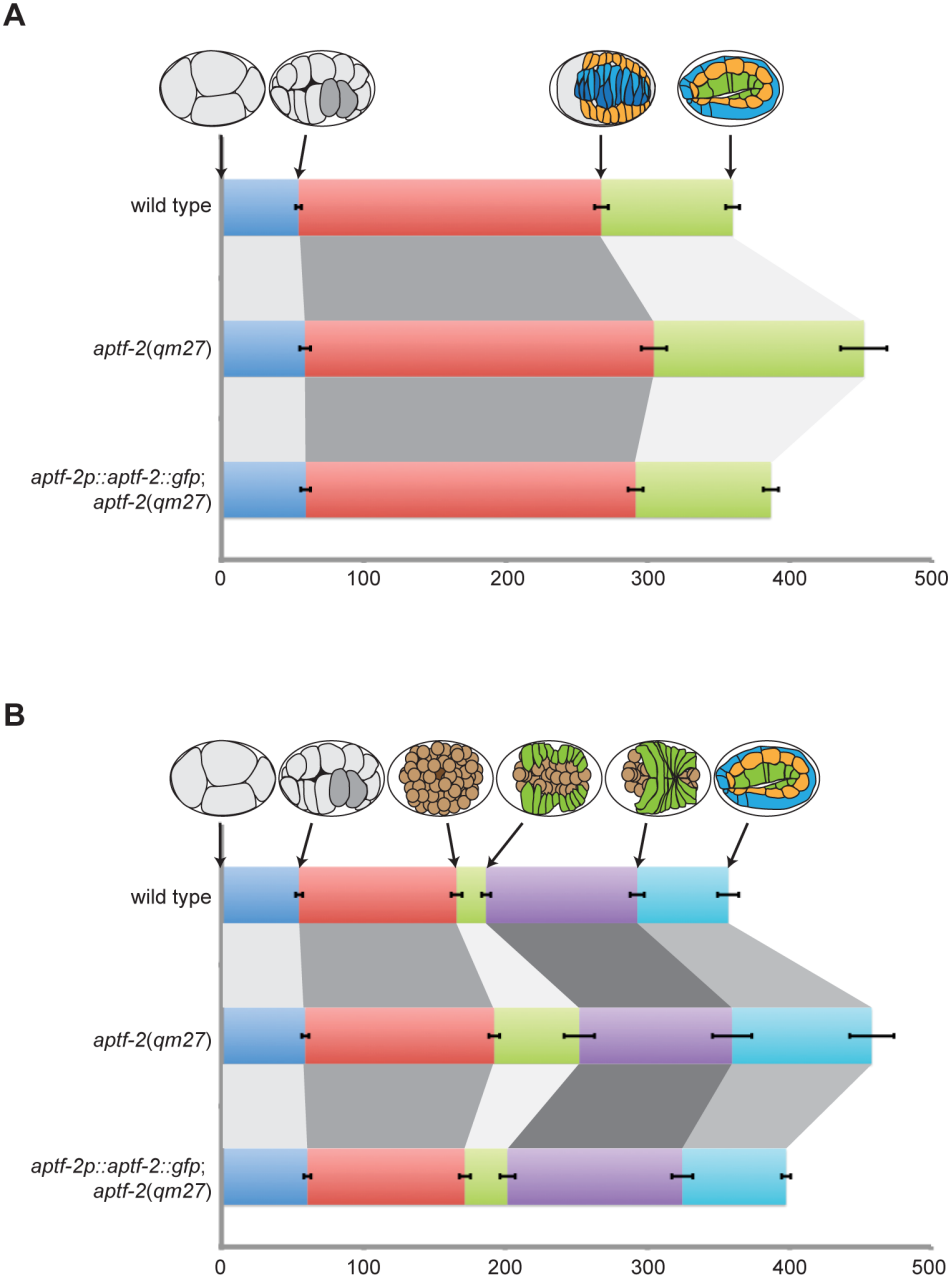
Embryogenesis is delayed in *aptf-2*(*qm27*) embryos. (A) Four milestone events are used to measure the progression through embryogenesis in the dorsally-oriented embryos: interphase of four-cell stage, Ea/p gastrulation, dorsal epidermal cell intercalation and elongation at the two-fold stage. Embryogenesis is delayed in *aptf-2*(*qm27*) embryos and this delay is rescued upon expression of APTF-2::GFP in the mutant background. (B) Progression through embryogenesis in the ventrally-oriented embryos is measured at six time points: interphase of four-cell stage, gastrulation of Ea/p cells, ventral cleft initiation, ventral cleft closure, epidermal ventral enclosure and elongation at two-fold stage. *aptf-2*(*qm27*) embryos progress slower through embryogenesis. Expression of APTF-2::GFP in *aptf-2*(*qm27*) embryos restores the embryogenesis timing almost to the wild-type level. Data are presented as mean ± s.e.m. n > 10 for each genotype. See S3 Table for the numerical values.

Analyzing the DIC movies of embryonic development we found that in addition to the various defects in epidermal morphogenesis the *aptf-2(qm27)* embryos developed more slowly than wild-type embryos at the same temperature. To quantify the delay and find out whether there is a particular stage in development that is slower or if all of embryogenesis is inherently slower we chose easy-to-recognize developmental milestones in dorsally or ventrally oriented embryos and measured the time it took for an embryo to progress from one milestone to the next (S3A and S3B Table). We also measured the same developmental times in *aptf-2(qm27)* embryos stably expressing wild-type APTF-2::GFP. The results, graphically presented in Fig 5, show that all stages of development are slower, to varying degrees, in *aptf-2(qm27)* embryos, and the developmental timing is mostly rescued in embryos ectopically expressing APTF-2::GFP. Specifically, ventral cleft closure is three times slower and elongation to 2 fold stage is one and a half times slower, while early development until Ea/Ep ingression is only slightly slower.

### Cell lineaging uncovers aberrant cell divisions in ABarp, C and D lineages in *aptf-2*(*qm27*) embryos

**Fig 6:**
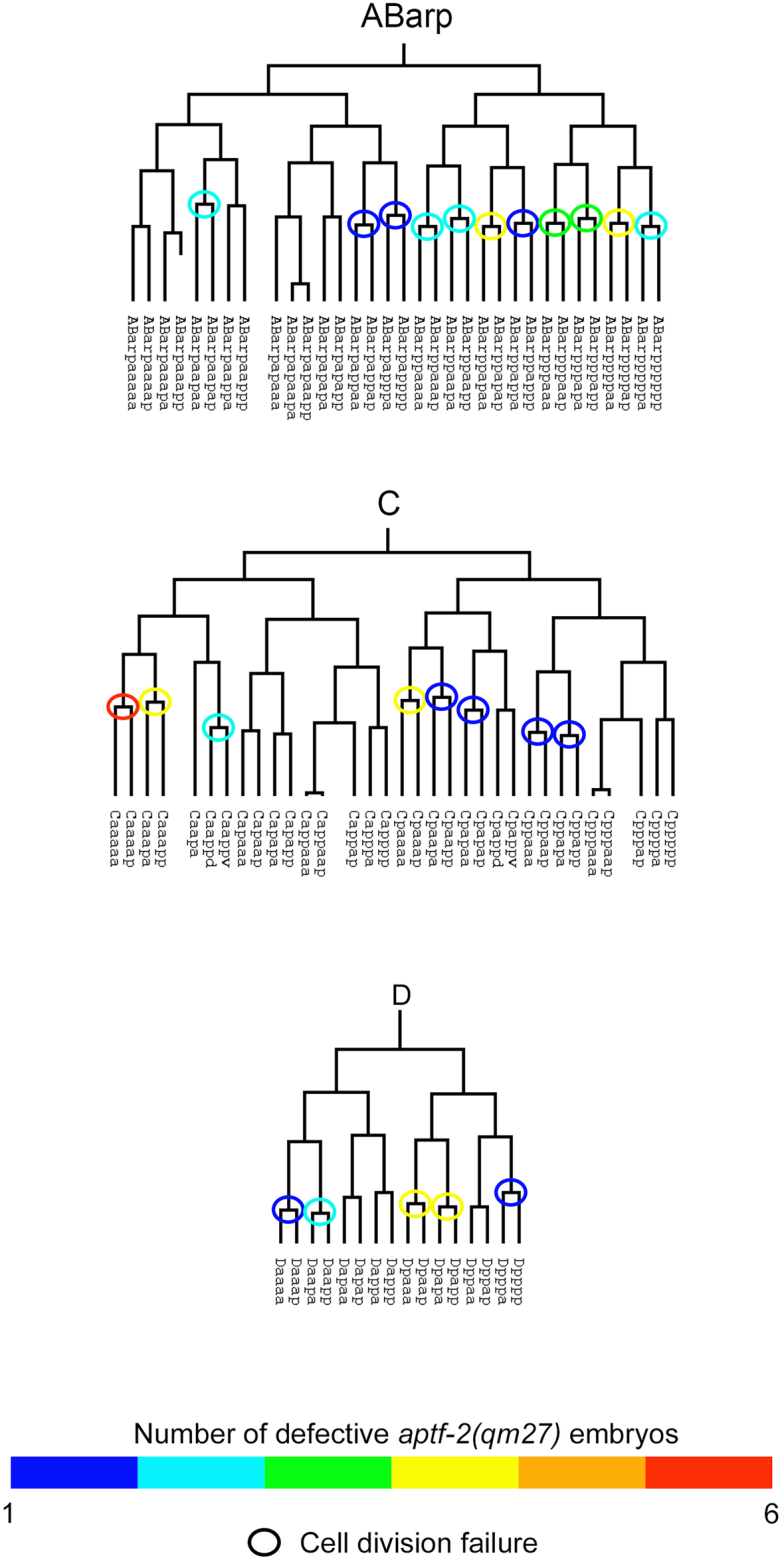
Aberrant cell division in ABarp, C and P4 lineages in *aptf-2*(*qm27*) mutant embryos. The cell division patterns in wild-type and *aptf-2*(*qm27*) embryos were examined using 4D microscopy and cell lineage analysis software (n = 2 for wild-type and n = 6 for *aptf-2*(*qm27*) embryos). Representative ABarp, C and D lineages for wild-type are shown. Circles mark defective cell division events while rectangles indicate apoptotic events in *aptf-2(qm27)* mutants. The markings are color-coded to illustrate the frequency of occurrence in the mutant embryos. See S4 Table for a complete list of names and fates of the cells that are defective in the *aptf-2*(*qm27*) embryos and S2a–S2m Fig for the complete lineage of all the six *aptf-2*(*qm27*) embryos.

To better understand the developmental defects in *aptf-2(qm27)* embryos we performed cell lineage analysis by following a nuclear marker, HIS-72::GFP, using 4D fluorescence microscopy. The cell division patterns in wild-type and *aptf-2*(*qm27*) embryos were captured, then analysed and edited using StarryNite and AceTree, respectively (n = 2 for wild-type and n = 6 for *aptf-2*(*qm27*) embryos). Cell division defects were consistently detected in three lineages: ABarp, C and D (Fig 6). The color markings drawn on the wild-type lineage trees illustrate the frequency of defects that occurred in the six *aptf-2*(*qm27*) mutant embryos analysed. Strikingly, failure in *aptf-2*(*qm27*) cell division occurs mostly in three lineages: ABarp, C and D with the Caaaa division absent in all six *aptf-2*(*qm27*) embryos analysed. The missing divisions resulted in the absence of epidermal seam cells and neuroblasts in the AB lineage and the absence of epidermal cells from the main body syncytium (hyp7), body wall muscle cells in the C and most of the D lineage (Fig 6 and S4 Table). In other cell lineages cell divisions appeared to be normal, except for an occasional division absent in the ABala or MSa lineages (S2–S14 Figs).

### APTF-2::GFP is enriched inside the nuclei of neuroblasts during the ventral cleft closure and inside the nuclei of dorsal epidermis preceding dorsal intercalation

**Fig 7:**
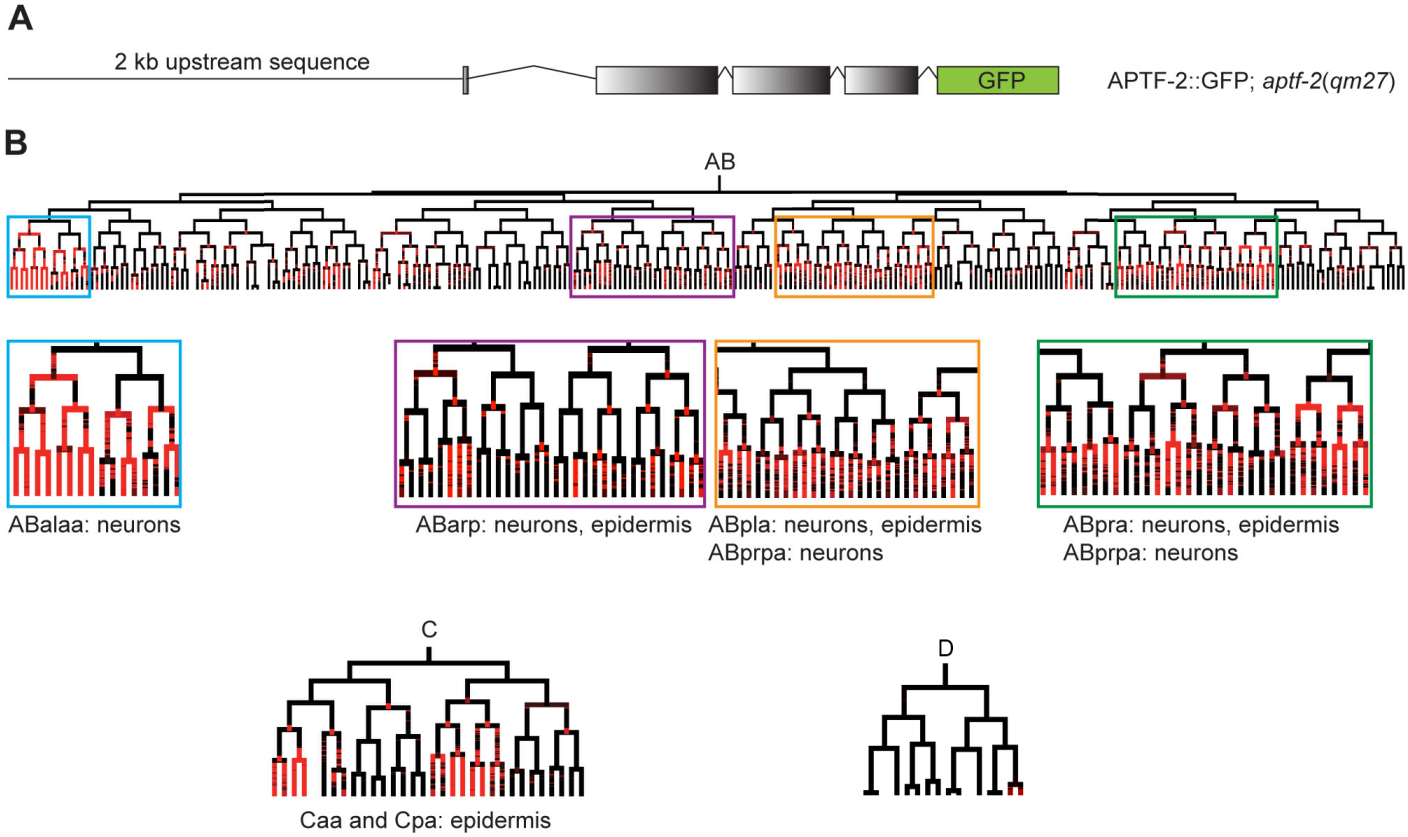
APTF-2::GFP is enriched in the nuclei of neuroblasts and epidermal cells. (A) An integrated construct consists of 2 kb *aptf-2* promoter followed by the *aptf-2* coding region tagged with GFP was co-expressed with HIS::mCherry in *aptf-2*(*qm27*) mutant worms and used for lineaging. (B) Representative AB, C and D lineages marking nuclear enrichment of APTF-2::GFP above a threshold is indicated in red color. See S15 Fig for the complete lineage of this embryo analysed for nuclear enrichment.

We used embryos co-expressing HIS::mCherry and the translational fusion of APTF-2::GFP driven by the *aptf-2* promoter to follow the subcellular localization of APTF-2 in specific cells during embryogenesis (Fig 6A). We found that in most cells APTF-2 is found uniformly in the nucleus and the cytoplasm. However, in certain cells at specific times during development, APTF-2 was enriched within the nucleus. Based on the lineaging of two embryos for 210 minutes we found significant nuclear enrichment of the APTF-2::GFP signal in neuroblasts and epidermal cells in AB lineage during ventral cleft closure and in epidermal cells in C lineage preceding dorsal intercalation (Fig 7B and S15 Fig). However, there does not appear to be a strong correlation between nuclear enrichment of APTF-2 and defects in cell division. While a high degree of nuclear enrichment was found in the C and ABarp lineages, in which the absence of cell division in *aptf-2(qm27)* embryos occured in 6/6 embryos, a high degree of nuclear enrichment was also found in ABpra and ABpla lineages that did not experience any defects in cell division. Similarly, in the D lineage, which did not show much nuclear enrichment, the failure in cell division was frequently observed.

### Aberrant nuclear localization does not explain the functional defects of the *qm27* allele of APTF-2

**Fig 8:**
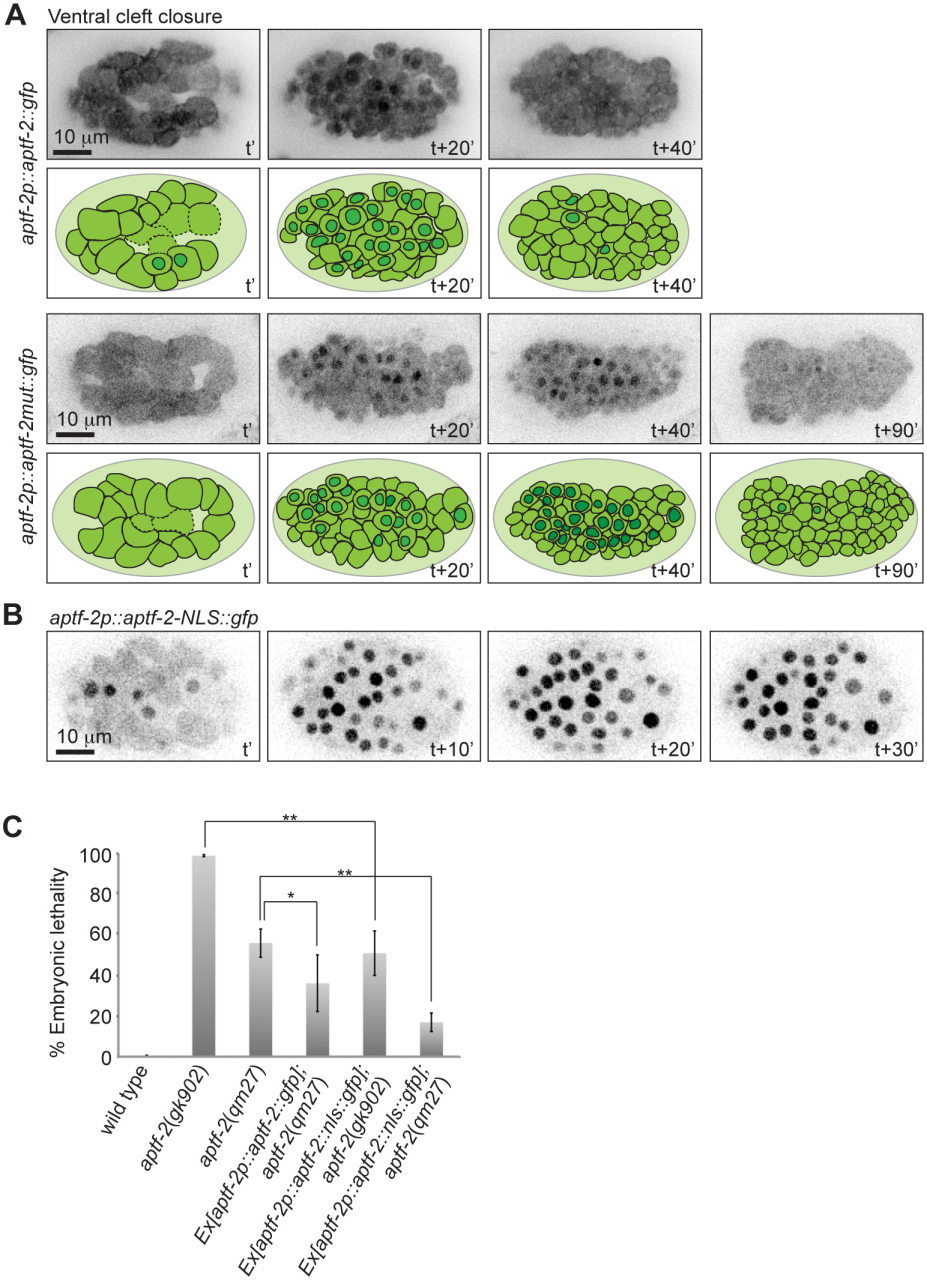
Nuclear retention of APTF-2 with *qm27* mutation does not explain its functional defect. (A) Mutated APTF-2 is also enriched inside the neuroblast nuclei during ventral cleft closure. However, the mutated APTF-2 stays longer inside the nucleus. Cartoons illustrating the embryos and highlighting the nuclear and the cytoplasmic localization of APTF-2 inside the cells are provided under each panel. t’ marks the start of the ventral cleft and t+40’ marks the completion of the ventral cleft closure. (B) Nuclear localization signal (NLS)-tagged APTF-2 is enriched inside nuclei of all cells throughout embryogenesis. t’ marks mid gastrulation. (C) Expression of APTF-2::NLS::GFP in *aptf-2* mutant worms rescues their embryonic lethality. Data are presented as mean ± s.e.m. n > 500 embryos for five independent experiments (*P<0.05, **P<0.01, two-tailed test). See S5 Table for the numerical values and statistical analysis.

In light of the specific nuclear enrichment of APTF-2 in the cell lineages where we observed defects in cell division timing in the *aptf-2(qm27)* hypomorph, we wondered whether the mutant protein has a defect in nuclear enrichment. To test this possibility we introduced into the APTF-2::GFP construct the same point mutation present in the *qm27* allele. As shown in Fig 8A, the mutant protein had no problem in becoming enriched in neuroblast nuclei during ventral cleft closure. To the contrary, once the mutant APTF-2 entered the nucleus, it appeared to remain enriched in the nucleus for longer than the wild-type protein. This raised the question whether abnormal nuclear retention of APTF-2 could explain the defects in *aptf-2(qm27)*. To address this question we engineered an APTF-2::GFP flanked by two nuclear localization signals from SV40 and EGL-13 and expressed it in *aptf-2(qm27)* and *aptf-2(gk902)* embryos. In contrast with wild-type APTF-2::GFP, APTF-2::NLS::GFP was continuously and exclusively nuclear in all cells in which it was expressed (Fig 8B). Importantly, expression of the constitutively nuclear APTF-2 was able to significantly rescue embryonic lethality of *aptf-2(qm27)* and *aptf-2(gk902)* (Fig 8C and S5 Table). These findings suggest that the aberrant nuclear localization of mutated APTF-2 does not explain its functional defects.

### APTF-4 cooperates with APTF-2 to regulate epidermal morphogenesis

**Fig 9:**
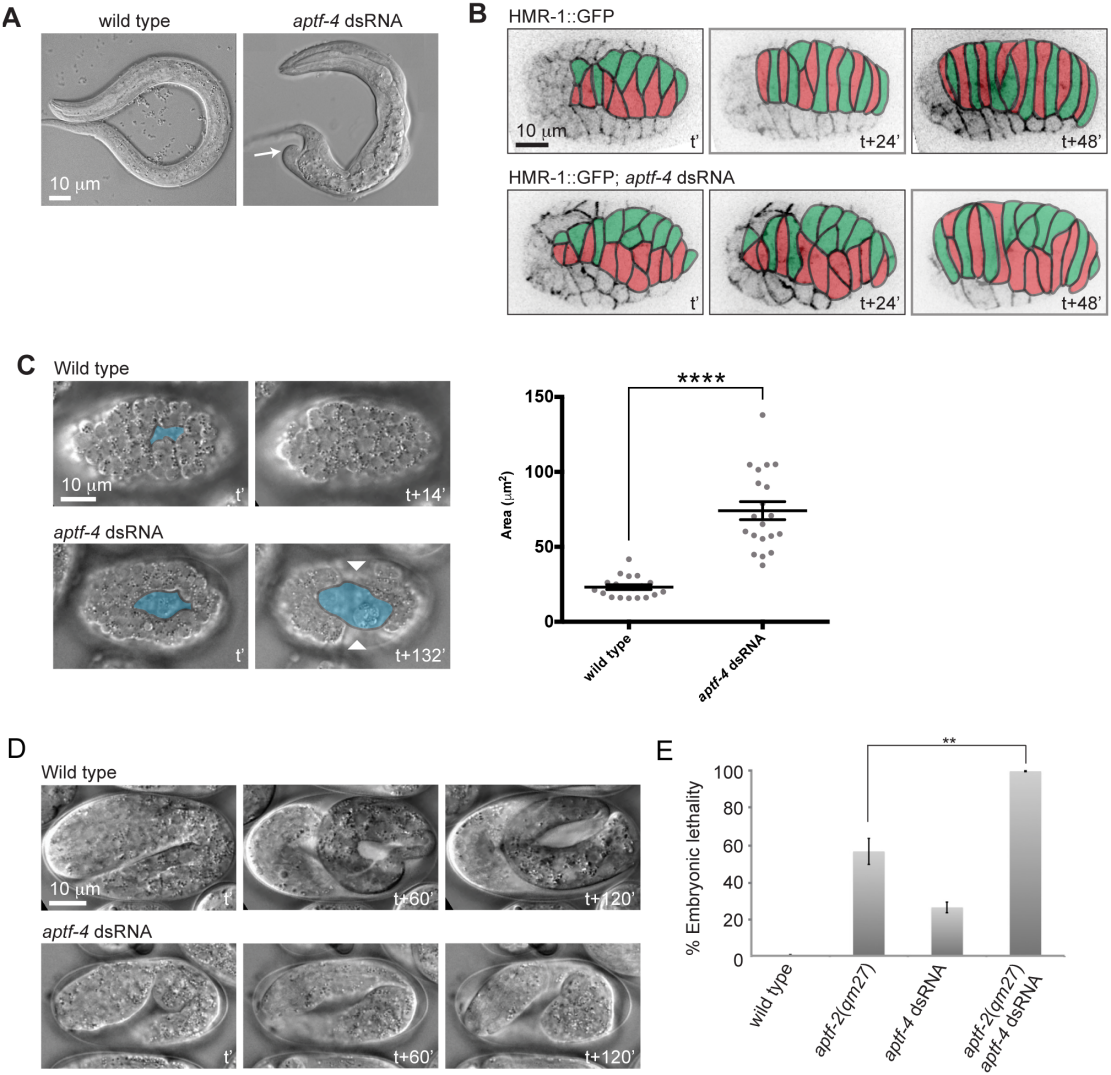
APTF-4 cooperates with APTF-2 in regulating epidermal morphogenesis. (A) L1 larva of wild-type and *aptf-4* dsRNA-treated L1 larva exhibiting a body morphology defect (indicated by arrow). (B) The intercalation of dorsal epidermal cells is defective in *aptf-4* dsRNA-treated embryos. Dorsal epidermal cells from two opposite rows are differently colored, in green and red, to better visualize the intercalation process. Cell junctions are visualized by HMR-1::GFP. t’ marks the timing of the two rows of epidermal cells aligning at the embryo dorsal side and ready to undergo intercalation. t+48’ marks the end of the intercalation process in wild type embryo. See corresponding S4 Movie. (C) Some *aptf-4* dsRNA-treated embryos have a relatively large ventral cleft that fails to close up before ventral enclosure takes place and thus results in defective ventral enclosure. Ventral cleft is colored in blue. t’ marks the first appearance of the ventral cleft and t+14’ marks its closure in wild type. In the quantification, error bars denote mean ± s.e.m. and p≤0.0001. Values for wild type is identical to that from Fig 3C. (D) *aptf-4* dsRNA-treated embryo is arrested during elongation. t’ marks elongation at two fold stage, whereas t+120’ marks elongation at 3.5 fold stage in wild type embryo. (E) The amount of embryonic lethality upon co-depletion of *aptf-2* and *aptf-4* is the sum of the embryonic lethality of *aptf-2* and *aptf-4* single depletion, suggesting cooperation between the two AP-2 transcription factors in regulating embryogenesis. Data are presented as mean ± s.e.m. n > 500 embryos for five independent experiments. (*P<0.05, **P<0.01, two-tailed test). See S6 Table for the numerical values and statistical analysis.

The worm genome encodes for four AP2-like transcription factors (S16 Fig). APTF-1 is expressed in only five head interneurons and is required for a sleep-active neuron to induce lethargus in molting larvae [21]. To test whether APTF-3 and/or APTF-4 may play a role in embryonic development we depleted zygotic and maternal products of the genes by RNAi and tested for embryonic lethality in the progeny. Knockdown of *aptf-3* did not result in any embryonic lethality. In contrast, knockdown of *aptf-4* resulted in 26 ± 3% embryonic lethality. Moreover, hatched *aptf-4(RNAi)* larvae often exhibited body morphology defects reminiscent of the defects observed in *aptf-2* mutants (Fig 9A). The deletion allele *aptf-4(gk582)* resulted in 100% larval arrest of homozygous worms, precluding analysis of embryonic phenotypes. Closer examination of embryonic development by 4D DIC and fluorescence microscopy revealed defects in dorsal intercalation, ventral cleft closure, and elongation (Fig 9B–9D, S4 Movie). To test whether APTF-2 and APTF-4 work independently or cooperatively in the regulation of epidermal morphogenesis we tested the combined effect of *aptf-4* KD in the background of *aptf-2(qm27)*. We found the embryonic lethality upon co-depletion of *aptf-2* and *aptf-4* to be higher than the sum of the lethality of single depletions, suggesting synergy between *aptf-2* and *aptf-4* (Fig 8E and S6 Table). As AP-2 transcription factors are believed to function as heterodimers in some cases [26], one possibility is that *aptf-2* and *aptf-4* work cooperatively. 4D DIC movie analysis revealed that 100% of the dorsally oriented dual-depleted embryos had dorsal intercalation defects and arrested during elongation and 57% of the ventrally oriented dual-depleted embryos displayed ventral cleft closure defects and 100% of them arrested in elongation (S7 Table). We used expression data for APTF-4 from the EPIC dataset (http://epic.gs.washington.edu/) to compare the nuclear expression pattern between APTF-2::GFP and APTF-4::GFP (S17 Fig). Both APTF-2::GFP and APTF-4::GFP showed similar nuclear enrichment in the AB and C lineages, consistent with their cooperativity in embryogenesis.

## Discussion

Vertebrates and *C*. *elegans* AP-2 TF genes share high sequence similarities in their functional domains, although the duplications leading to four family members appear to have occurred independently in *C*. *elegans* and in vertebrates (S16 Fig). In this study, we report that partial loss of *aptf-2* or *aptf-4* resulted in body morphological defects. Patients with BOFS suffer from skin defects while complications associated with Char Syndrome result from derangement of neural-crest-cell derivatives [17, 18]. Our findings from the characterization of *aptf-2(qm27)* share similarity with the pathological manifestation of BOFS and Char Syndrome patients in epidermal and neuronal tissues. The mutation in the *aptf-2(qm27)* allele lies in the basic region of the DNA binding domain, a region that was defined as a mutation hotspot for BOFS and Char Syndorme in the human TFAP2A and TFAP2B genes [17–20]. At least 24 mutations in the basic region have been identified for BOFS and five for Char Syndrome [18, 20, 27]. It is challenging to determine the genotype-phenotype relationship in BOFS and Char Syndrome patients due to the small sample size and the large spectrum of mutations affecting *TFAP2A* and *TFAP2B*. With recent advances in site-targeted mutagenesis in the *C*. *elegans* genome, it is an exciting possibility to generate worm strains carrying mutations of conserved residues in BOFS and Char Syndrome.

The *aptf-2(gk902)* allele results in a frame shift, generating a null allele. The massive apoptotic phenotype observed following a complete loss of APTF-2 in *aptf-2(gk902)* embryos is drastically different from the epidermal morphogenesis defects observed when APTF-2 activity is partially compromised as with the *aptf-2(qm27)* allele. This suggests different thresholds of AP-2 transcriptional activity are required for different cellular functions. Interestingly, in Char Syndrome patients, hypomorphic mutations in *TFAP2B* result in congenital heart defect, whereas a complete deletion of the mouse ortholog, AP-2β, leads to polycystic kidney disease due to excessive apoptosis of renal epithelial cells [14, 18].

In murine models, depletion of AP-2γ resulted in defective epidermal development due to delayed expression of epidermal differentiation genes [28]. This is consistent with our observation that *aptf-2* mutants showed epidermal morphogenesis defects. Neural crest defects in mouse, zebrafish and *Xenopus* embryos have been attributed to loss of AP-2 transcription factors [1, 29, 30], parallel to the neuroblast migration defect we observed in the *C*. *elegans* embryo. Earlier expression studies of AP-2 transcription factors were largely conducted in mice, *Drosophila* and *Xenopus* by observing in-situ hybridization and staining patterns [5, 16, 31–33]. Our work in the live *C*. *elegans* embryo provided spatio-temporal information at a resolution not described previously. We observed APTF-2::GFP to be enriched in the nuclei of neuroblasts and epidermal cells during ventral enclosure and dorsal intercalation respectively, lack of which (in the case of the mutant) resulted in aberrant cell division in the epidermal and neuroblast lineages. Thus, our work identified lineage-specific regulation of cell division timing by APTF-2. Similar mechanisms could be at play in mammals. Interestingly, we observed that nuclear enrichment of APTF-2 does not always correlate with regulation of cell division, as in the case of D, suggesting that a lower level of nuclear APTF-2 may be required for the division in this lineage. In contrast, nuclear APTF-2 enrichment was observed in ABpra and ABpla and yet an absence of cell division was not been observed in these lineages in *aptf-2*(*qm27*) embryos, indicating that either a stronger APTF-2 depletion is required to see cell division defects or APTF-2 plays a different role in these two lineages.

Although various members of the vertebrate AP-2 transcription family have been shown to have overlapping expression patterns, knockout studies in mice revealed specific and localized phenotypic defects. For example, Moser *et*. *al*. showed that the AP-2α and AP-2β expression in mouse embryos overlap significantly, [16], but the single knockout models of each gene did not share any phenotypic defects, suggesting non-redundant roles of the two genes [14]. In contrast to the vertebrate system, our results showed both similar phenotypes and similar expression pattern, mostly in AB and C lineages of *aptf-2* and *aptf-4* in the worm. The fact that their effect is synergistic suggests they may partially function through the same pathway.

For wild-type APTF-2::GFP, expression in the majority of cells was evenly distributed between the nucleus and cytoplasm and was enriched in the nucleus of neuroblasts during ventral cleft closure and in epidermal cells preceding dorsal intercalation. It is possible that APTF-2 functions to regulate gene expression at a basal level, while enrichment in the nucleus of specified cells during epidermal morphogenesis upregulates genes required for proliferation of the neuroblasts and epidermal cells. This would be consistent with observations in *Drosophila*, where different levels of AP-2 have been shown to result in a variety of morphological defects [32].

AP-2 transcription factors are known to play a dual role as transcription activators and repressors [33]. Pfisterer *et*. *al*. identified multiple genes repressed by AP-2α known to induce apoptosis and retards proliferation [34]. There has also been evidence in *Xenopus* epidermal development regarding the importance of AP-2 TF in promoting the expression of epidermal specific genes [31]. We used TargetOrtho to identify putative APTF-2 targets. Among the candidates, we tested *die-1*, a well known regulator of epidermal dorsal intercalation, and observed the reduction of DIE-1 nuclear signal in *aptf-2*(*qm27*) embryos, suggesting that DIE-1 is likely a target of APTF-2. Future work must determine APTF-2 target genes in neuroblasts and epidermal cells in order to further elucidate its function during morphogenesis.

In conclusion, we have characterized a hypomorphic mutant of *C*. *elegans* APTF-2 and have shown it to share genetic and anatomical similarities with human Char Syndrome and Bronchio-occular-facial Syndrome. We propose mutations in *C*. *elegans* AP-2 TF genes can serve as disease models to study the cellular mechanisms and tissue dynamics that lead from mutant genotype to disease phenotype.

## Materials and Methods

### Strains and alleles

Strains were maintained at 20°C under standard conditions [35]. Wild-type Bristol strain N2 was used as a control. The *aptf-2*(*qm27*) *IV* line was retrieved in an EMS screen conducted by Hekimi et al. [22] and *aptf-2*(*gk902*) was generated by the *C*. *elegans* Reverse Genetics Core Facility at the University of British Columbia and was maintained as heterozygotes using the *nT1[qIs51] (IV;V)* balancer. For analysis using GFP reporters, F_2_ progeny exhibiting *aptf-2* phenotypes and carrying the markers were selected from crosses between *aptf-2*(*qm27*) and the following strains: FT250 *xnIs96 [pJN455(hmr-1p*::*hmr-1*::*GFP*::*unc-54 3'UTR) + unc-119(+)]* [36], SU265 *jcIs17[hmp-1p*::*hmp-1*::*gfp*, *dlg-1p*::*dlg-1*::*dsRed*, *rol-6p*::*rol-6(su1006)]* [37], OH904 *otIs33[kal-1p*::*gfp]* [38], RW10029 *zuIs178 [his-72(1kb 5' UTR)*::*his-72*::*SRPVAT*::*GFP*::*his-72 (1KB 3' UTR) + 5*.*7 kb XbaI—HindIII unc-119(+)]*. *stIs10024 [pie-1*::*H2B*::*GFP*::*pie-1 3' UTR + unc-119(+)]* (a gift from Waterston lab) and JIM119 *zuIs178 [his-72(1kb 5' UTR)*::*his-72*::*SRPVAT*::*mCherry*::*his-72 (1KB 3' UTR) + 5*.*7 kb XbaI—HindIII unc-119(+)]*. *stIs10024 [pie-1*::*H2B*::*mCherry*::*pie-1 3' UTR + unc-119(+)]* (a gift from Waterston lab). *die-1*::*gfp* reporter strain was a gift from Hardin lab [25].

### Plasmid construction

To construct plasmids containing wild-type or mutated *aptf-2*, the *aptf-2* promoter (2 kb sequence upstream of *aptf-2* start codon) followed by the *aptf-2* coding sequence were amplified from N2 and *aptf-2*(*qm27*) animals, respectively and inserted into *Xba*I and *Age*I sites upstream of *gfp* in the original pPD95.75 vector. The wild-type *aptf-2*-containing plasmid was injected into the gonad of *aptf-2*(*qm27*) hermaphrodite animals to examine its potency in rescuing *aptf-2*(*qm27*) phenotypes, whereas the plasmid containing mutated *aptf-2* was injected into N2. This resulted in the following transgenes: *msnEx15* [*aptf-2p*::*aptf-2*::*gfp*; *rol-6*(*su1006*)]; *aptf-2*(*qm27*) and *msnEx239* [*aptf-2p*::*mutated aptf-2*::*gfp*; *rol-6*(*su1006*)]. Ten L4 larvae expressing wild-type *aptf-2* were subjected to a UV source (BioRad) for 15 seconds to integrate the extrachromosomal array into the genome. Three hundred F_2_ worms were then singled and incubated for three weeks and subsequently examined for expression and embryonic lethality. Those expressing the transgene and giving rise to 100% viable progeny were selected and outcrossed. The resulting strain, RZB104 (*aptf-2*(*qm27*); *msnIn104*[*aptf-2p*::*aptf-2*::*gfp*; *rol-6*(*su1006*)]), was used throughout this study.

To construct *aptf-2* tagged with a nuclear localization signal (NLS), the amplified 4.3 kb genomic sequence containing the *aptf-2* promoter and the coding region was inserted into *Xba*I and *Xma*I sites in pNL74.4 [39], a modified pPD95.75 containing SV40 and EGL-13 NLS flanking the N and the C terminal of the gfp sequence, respectively. The plasmid was injected into the gonad of N2 hermaphrodites and resulted in transgene *msnEx103* [*aptf-2p*::*aptf-2-NLS*::*gfp*; *rol-6*(*su1006*)]). The transgenic animals were then crossed with *aptf-2*(*qm27*) or *aptf-2*(*gk902*) to assess the ability of NLS-tagged APTF-2 to rescue the *aptf-2* mutants.

### Microinjection

Microinjection was performed as described by Mello and Fire [40]. Injection mix included 100 μg/μl salmon sperm DNA digested with *Pvu*II, 20 μg/μl *rol-6*(*su1006*) digested with *Sbf*I and 5–10 μg/μl each construct digested with *Sbf*I.

### Whole genome sequencing and mutation validation

Genomic DNA was extracted from *mal-1*(*qm27*) mutant worms using standard method and subjected to whole genome sequencing using Illumina platform and annotated using MAQGene [41]. The whole genome sequencing and its annotation were performed by Hobert lab (Columbia University). Candidate genes altered in *mal-1*(*qm27*) were narrowed down using genetic mapping results done by Hekimi et al. [22]. Point mutation in *aptf-2* gene was confirmed by amplification of *aptf-2* gene in *aptf-2*(*qm27*) mutant worms, subcloning into pJET vector (Thermo Scientific) and followed by conventional sequencing (First Base).

### Complementation assay, brood size analysis and larva phenotype scoring

For complementation assay, *aptf-2(gk902)/nT1[qIs51]* males was crossed with *aptf-2*(*qm27*) hermaphrodites. Non-GFP F_1_ animals were singled and incubated to lay embryos for 24 hours. The F_1_ animals were genotyped for the *gk902* deletion and only the cross progeny between *qm27* and *gk902* alleles was scored for embryonic lethality of their F_2_s.

For brood size analysis, ten L4 larvae of wild-type, *aptf-2*(*qm27*) and *aptf-2*(*gk902*) were singled and incubated for 24 hours. Each animal was shifted to a new plate every day for 5 consecutive days to the point that no more embryos were laid. The total number of embryos laid was determined as the brood size. The number of hatched animals was calculated and used to determine the percentage of embryonic lethality. Larvae that did not grow into adult in 48–92 hours after hatching were considered as being arrested. *aptf-2*(*qm27*) and *aptf-2*(*gk902*) larvae of any stage were subjected to phenotypic analysis to determine the presence and the position of the morphological defects.

### Quantification of embryonic lethality

Besides wild-type, *aptf-2*(*qm27*) and *aptf-2*(*gk902*) animals whose embryonic lethality was determined as described above, the embryonic lethality of the remaining strains were determined as follows: ten to fifteen gravid hermaphrodites were placed on the plate and incubated at 20°C for several hours to lay more than 100 embryos. Hermaphrodites were then removed and the number of embryos laid was counted. Twenty-four hours later, the number of larvae hatched was determined. Each experiment was repeated at least five times.

### 4D microscopy

Larvae or embryos collected from gravid hermaphrodite were mounted onto 3% agarose-padded glass slide, closed with a coverslip and sealed with wax. DIC images shown in Figs 1A, 3A, 3B, 3C, 8A, 8C, 8E and S1A were captured using a Nikon Ti Eclipse widefield microscope equipped with DIC 1.40NA oil condenser and a charged-coupled device camera Cool Snap HQ_2_ (Photometrics). All other images and movies were acquired using a spinning disk confocal system composed of a Nikon Ti Eclipse microscope with a CSU-X1 spinning disk confocal head (Yokogawa), DPSS-Laser (Roper Scientific) at 491 and 568 nm excitation wavelengths and an Evolve Rapid-Cal electron multiplying charged-coupled device camera (Photometrics). For both microscopes, Metamorph software (Molecular Devices) was used to control acquisition. Projected images were created using Fiji. All imaging was done at 20°C in an environmental chamber encompassing the microscope stage heated by a JCS temperature controller (Shinko Technos Co, Japan) within a microscope room kept at 18°C by a CITEC precision air conditioning unit.

### Knockdown experiments

*aptf-4* dsRNA was synthesized as described [42] and injected into the gonad of twenty wild-type or *aptf-2*(*qm27*) L4 larvae. Each animal was singled into a separate plate and its embryonic lethality was examined 24 hours post injection.

### Bioinformatics

Protein sequence of the AP-2 transcription factor family members in the following metazoan species were aligned using Constraint-based Multiple Protein Alignment Tool (COBALT) [43]: *A*. *queenslandica* (sponge), *T*. *adhaerens* (Placozoa), *C*. *elegans* (nematode), *N*. *vectensis* (sea anemone), *D*. *melanogaster* (fruit fly), *S*. *purpuratus* (sea urchin), *C*. *intestinalis* (tunicate), *B*. *floridae* (lancelete), *D*. *rerio* (fish), *X*. *tropicalis* (frog), *G*. *gallus* (chicken), *H*. *sapiens* (human). The resulting alignment was used to build and visualize a phylogenetic tree (neighbor-joining method) using *Geneious* (Biomatters Ltd.). Illustration of the gene and protein architecture was drawn using Illustrator for Biological Sequences [44].

### Predicting AP2-TF target genes using TargetOrtho

AP-2 has been shown to bind to the palindromic consensus sequence 5'-GCCN3GGC-3', as well as the binding motif 5'-GCCN3/4GGG-3' [2]. We used either the 9bp or 10bp motif as an input for TargetOrtho [23]. From the program output we selected only putative targets that are conserved in at least 4 *Caenoharbditis* species, and are located within the 3 kb region upstream of the start codon. Functional annotation was performed using DAVID Bioinformatics Resources 6.7 [45, 46] and the threshold we used for enrichment was an EASE score equal or smaller than 0.05.

### Cell lineaging

For cell lineaging, six *aptf-2*(*qm27*) embryos expressing nuclear signal of GFP::HIS-72 and two embryos co-expressing APTF-2::GFP and mCherry::HIS-72 were analysed for at least 270 minutes according to the protocol described in [47–49]. The lineage tree was built using AceTree [50] and compared to that of wild-type. To visualize the temporal enrichment of the nuclear APTF-2::GFP signal during embryogenesis, the minimum/ maximum threshold values were set to display the 75% highest signal. All movies used for lineaging in this paper can be downloaded from http://epic2.gs.washington.edu/Epic2.

### Statistical analysis

Statistical analyses were done using Prism 6 (GraphPad Software, La Jolla, CA). Two-tailed Student’s *t*-test was applied to compare the values.

## Supporting Information

S1 Fig A neuroblast migration defect precedes the ventral epidermal cell enclosure defect in *aptf-2*(*qm27*) embryos. (A) L4 of wild type and *aptf-2(qm27)* mutants visualized by DIC microscopy. (B) Gastrulation cleft closure in wild-type and *aptf-2(qm27)* embryos visualized by DIC microscopy. Ventral clefts are colored in blue and white arrows indicate ventral enclosure process. In the quantification of cleft size, error bar denotes mean ± s.e.m., p≤0.0001. (C) Ventral neuroblast migration to the midline in a wild-type embryo and their failure to migrate in an *aptf-2*(*qm27*) embryo. A maximum intensity projection of KAL-1::GFP expression is used to visualize neuroblasts. Red arrows indicate the progression of ventral cleft closure.
(TIF)

S2 Fig ABala lineage of wild-type and six *aptf-2(qm27)* embryos. *aptf-2(qm27)* embryos 1–3 are lineaged to 315 minutes, *aptf-2(qm27)* embryo 4 to 295 minutes, wild type, *aptf-2(qm27)* 5 and 6 are lineaged to 270 minutes respectively. Defects in cell division are marked with an X.
(TIF)

S3 Fig ABalp lineage of wild-type and six *aptf-2(qm27)* embryos. *aptf-2(qm27)* embryos 1–3 are lineaged to 315 minutes, *aptf-2(qm27)* embryo 4 to 295 minutes, wild type, *aptf-2(qm27)* 5 and 6 are lineaged to 270 minutes respectively.
(TIF)

S4 Fig ABpla lineage of wild-type and six *aptf-2(qm27)* embryos. *aptf-2(qm27)* embryos 1–3 are lineaged to 315 minutes, *aptf-2(qm27)* embryo 4 to 295 minutes, wild type, *aptf-2(qm27)* 5 and 6 are lineaged to 270 minutes respectively.
(TIF)

S5 Fig ABplp lineage of wild-type and six *aptf-2(qm27)* embryos. *aptf-2(qm27)* embryos 1–3 are lineaged to 315 minutes, *aptf-2(qm27)* embryo 4 to 295 minutes, wild type, *aptf-2(qm27)* 5 and 6 are lineaged to 270 minutes respectively.
(TIF)

S6 Fig ABara lineage of wild-type and six *aptf-2(qm27)* embryos. *aptf-2(qm27)* embryos 1–3 are lineaged to 315 minutes, *aptf-2(qm27)* embryo 4 to 295 minutes, wild type, *aptf-2(qm27)* 5 and 6 are lineaged to 270 minutes respectively.
(TIF)

S7 Fig ABarp lineage of wild-type and six *aptf-2(qm27)* embryos. *aptf-2(qm27)* embryos 1–3 are lineaged to 315 minutes, *aptf-2(qm27)* embryo 4 to 295 minutes, wild type, *aptf-2(qm27)* 5 and 6 are lineaged to 270 minutes respectively. Defects in cell division are marked with an X.
(TIF)

S8 Fig ABpra lineage of wild-type and six *aptf-2(qm27)* embryos. *aptf-2(qm27)* embryos 1–3 are lineaged to 315 minutes, *aptf-2(qm27)* embryo 4 to 295 minutes, wild type, *aptf-2(qm27)* 5 and 6 are lineaged to 270 minutes respectively.
(TIF)

S9 Fig ABprp lineage of wild-type and six *aptf-2(qm27)* embryos. *aptf-2(qm27)* embryos 1–3 are lineaged to 315 minutes, *aptf-2(qm27)* embryo 4 to 295 minutes, wild type, *aptf-2(qm27)* 5 and 6 are lineaged to 270 minutes respectively.
(TIF)

S10 Fig MSa lineage of wild-type and six *aptf-2(qm27)* embryos. *aptf-2(qm27)* embryos 1–3 are lineaged to 315 minutes, *aptf-2(qm27)* embryo 4 to 295 minutes, wild type, *aptf-2(qm27)* 5 and 6 are lineaged to 270 minutes respectively.
(TIF)

S11 Fig MSp lineage of wild-type and six *aptf-2(qm27)* embryos. *aptf-2(qm27)* embryos 1–3 are lineaged to 315 minutes, *aptf-2(qm27)* embryo 4 to 295 minutes, wild type, *aptf-2(qm27)* 5 and 6 are lineaged to 270 minutes respectively.
(TIF)

S12 Fig E lineage of wild-type and six *aptf-2(qm27)* embryos. *aptf-2(qm27)* embryos 1–3 are lineaged to 315 minutes, *aptf-2(qm27)* embryo 4 to 295 minutes, wild type, *aptf-2(qm27)* 5 and 6 are lineaged to 270 minutes respectively. Defect in cell division is marked with an X.
(TIF)

S13 Fig C lineage of wild-type and six *aptf-2(qm27)* embryos. *aptf-2(qm27)* embryos 1–3 are lineaged to 315 minutes, *aptf-2(qm27)* embryo 4 to 295 minutes, wild type, *aptf-2(qm27)* 5 and 6 are lineaged to 270 minutes respectively. Defects in cell division are marked with an X.
(TIF)

S14 Fig D and P4 lineages of wild-type and six *aptf-2(qm27)* embryos. *aptf-2(qm27)* embryos 1–3 are lineaged to 315 minutes, *aptf-2(qm27)* embryo 4 to 295 minutes, wild type, *aptf-2(qm27)* 5 and 6 are lineaged to 270 minutes respectively. Defects in cell division are marked with an X.
(TIF)

S15 Fig Nuclear enrichment of APTF-2 during embryogenesis. The lineage of an embryo expressing APTF-2::GFP and HIS::mCherry was analyzed for nuclear enrichment of APTF-2::GFP. Nuclear enrichment is represented in red. APTF-2 is enriched in the AB and C lineages during embryogenesis at the time of ventral cleft closure and pre dorsal intercalation. The lineage was analysed to 232 minutes.
(TIF)

S16 Fig Phylogenetic tree of AP-2 transcription factor family across species. The amino acid sequences were aligned using Constraint-based Multiple Protein Alignment Tool (COBALT) and the phylogenetic tree was built using neighbor-joining method and visualized using *Geneious*. Species: *C*. *elegans* (soil worm), *A*. *queenslandica* (sponge), *T*. *adhaerens* (a simple metazoan), *N*. *vectensis* (sea anemone), *B*. *floridae* (lancelet), *D*. *melanogaster* (fruit fly), *C*. *intestinalis* (tunicate), *S*. *purpuratus* (sea urchin), *D*. *rerio* (fish), *G*. *gallus* (chicken), *X*. *tropicalis* (frog), *H*. *sapiens* (human). *C*. *elegans* APTF-2, which is the subject of this study is highlighted in blue.
(TIF)

S17 Fig Comparison between the nuclear expression pattern of APTF-2::GFP and APTF-4::GFP. Both lineage trees shows enriched expression in the AB and C lineages while the MS, E and D has weak expression. Trees were drawn to 210 minutes.
(TIF)

S1 Table Morphological defects of *aptf-2* mutant worms. (DOCX)

S2 Table Expression of APTF-2::GFP in *aptf-2*(*gk902*) and *aptf-2*(*qm27*) animals rescues their embryonic lethality. (DOCX)

S3 Table APTF-2 is required for timely embryogenesis events (A dorsally-oriented embryos B ventrally-oriented embryos). (DOCX)

S4 Table Affected lineages in *aptf-2*(*qm27*) mutant embryos. (DOCX)

S5 Table Nuclear targeting of APTF-2 is sufficient to rescue *aptf-2*(*gk902*) and *aptf-2*(*qm27*) embryonic lethality. (DOCX)

S6 Table APTF-2 and APTF-4 synergistically regulate *C*. *elegans* embryogenesis. (DOCX)

S7 Table Phenotypic analysis of *aptf-2*(*qm27*) embryos injected with *aptf-4* dsRNA and analyzed by DIC. (DOCX)

S1 Movie Time lapse DIC and spinning disk microscopy showing that the expression of APTF-2::GFP rescues *aptf-2*(*qm27*) embryonic lethality. Movie starts from early embryogenesis and ends at the time of hatching. Only a single focal plane of the embryo epidermis is shown. Time is indicated in minutes. Embryo at the bottom expresses APTF-2::GFP, whereas embryo at the top does not.
(AVI)

S2 Movie Dorsal epidermal cell intercalation in wild-type and *aptf-2*(*qm27*) embryos expressing HMR-1::GFP. Movie starts with two rows of dorsal epidermal cells ready to intercalate and ends with the wild-type embryo hatching and the *aptf-2(qm27)* embryo arrested during elongation. Time is indicated in minutes.
(AVI)

S3 Movie Ventral enclosure in wild-type and *aptf-2*(*qm27*) embryos expressing HMP-1::GFP. Movie starts when ventral epidermal cells initiate embryo enclosure and ends with the wild-type embryo hatching, whereas the mutant embryo is not properly enclosed, resulting in the internal tissues leaking out from the gap on the ventral side. Time is indicated in minutes.
(AVI)

S4 Movie Time lapse spinning disk microscopy showing dorsal epidermal cell intercalation in *aptf-4* dsRNA embryo expressing HMR-1::GFP. Movie starts with two rows of dorsal epidermal cells ready to intercalate. The intercalation is abnormal and ends with the embryo being arrested during elongation. Time is indicated in minutes.
(AVI)

S1 Text List of putative target genes of AP-2 transcription factors in the C. elegans genome generated by TargetOrtho (see materials and methods for details). (XLSX)

S2 Text zip files containing the nuclei files of *aptf-2(qm27)* mutant #1 produced by StarryNite [47] in a format readable by AceTree. (ZIP)

S3 Text zip files containing the nuclei files of *aptf-2(qm27)* mutant #2 produced by StarryNite [47] in a format readable by AceTree. (ZIP)

S4 Text zip files containing the nuclei files of *aptf-2(qm27)* mutant #3 produced by StarryNite [47] in a format readable by AceTree. (ZIP)

S5 Text zip files containing the nuclei files of *aptf-2(qm27)* mutant #4 produced by StarryNite [47] in a format readable by AceTree. (ZIP)

S6 Text zip files containing the nuclei files of *aptf-2(qm27)* mutant #5 produced by StarryNite [47] in a format readable by AceTree. (ZIP)

S7 Text zip files containing the nuclei files of *aptf-2(qm27)* mutant #6 produced by StarryNite [47] in a format readable by AceTree. (ZIP)
