# Supplementary figures and images for: The AP-2 Transcription Factor APTF-2 Is Required for Neuroblast and Epidermal Morphogenesis in Caenorhabditis elegans Embryogenesis
Source: PLoS Genet. 2016 May 13;12(5):e1006048. doi: 10.1371/journal.pgen.1006048 (PMC4866721; doi:10.1371/journal.pgen.1006048)

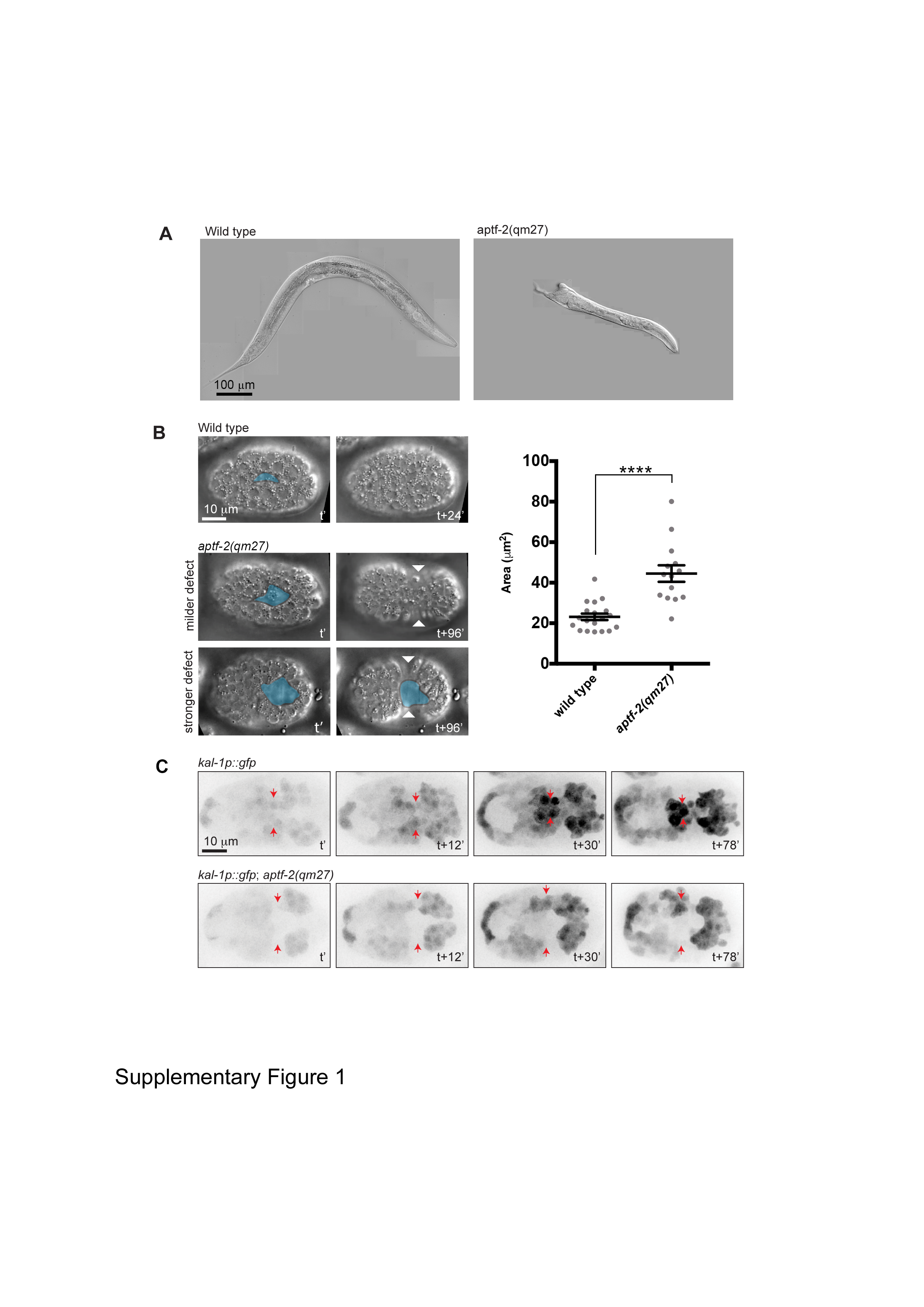

Supplement: S1 Fig — (A) L4 of wild type and aptf-2(qm27) mutants visualized by DIC microscopy. (B) Gastrulation cleft closure in wild-type and aptf-2(qm27) embryos visualized by DIC microscopy. Ventral clefts are colored in blue and white arrows indicate ventral enclosure process. In the quantification of cleft size, error bar denotes mean ± s.e.m., p≤0.0001. (C) Ventral neuroblast migration to the midline in a wild-type embryo and their failure to migrate in an aptf-2(qm27) embryo. A maximum intensity projection of KAL-1::GFP expression is used to visualize neuroblasts. Red arrows indicate the progression of ventral cleft closure. (TIF) [file pgen.1006048.s001.tif]

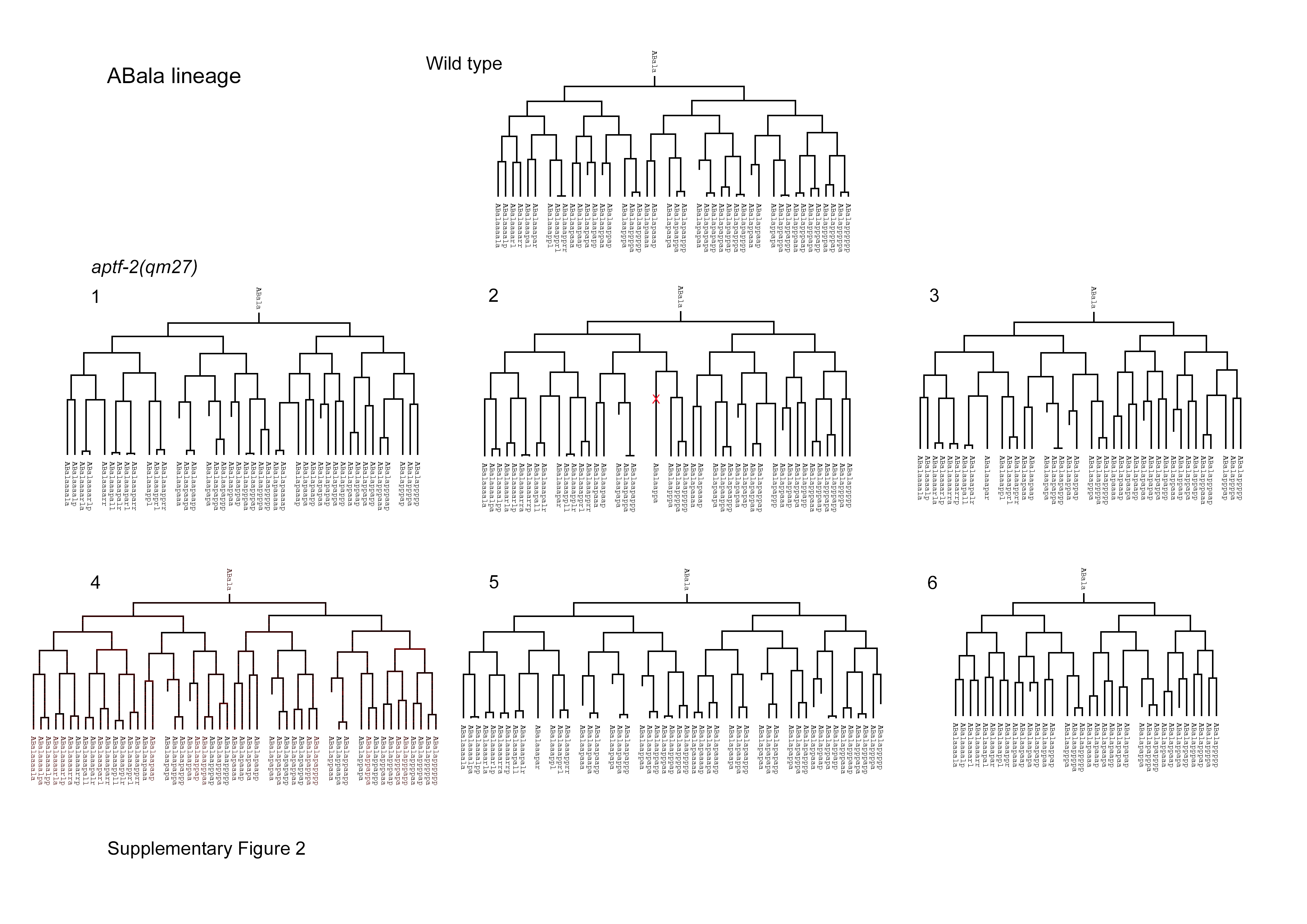

Supplement: S2 Fig — aptf-2(qm27) embryos 1–3 are lineaged to 315 minutes, aptf-2(qm27) embryo 4 to 295 minutes, wild type, aptf-2(qm27) 5 and 6 are lineaged to 270 minutes respectively. Defects in cell division are marked with an X. (TIF) [file pgen.1006048.s002.tif]

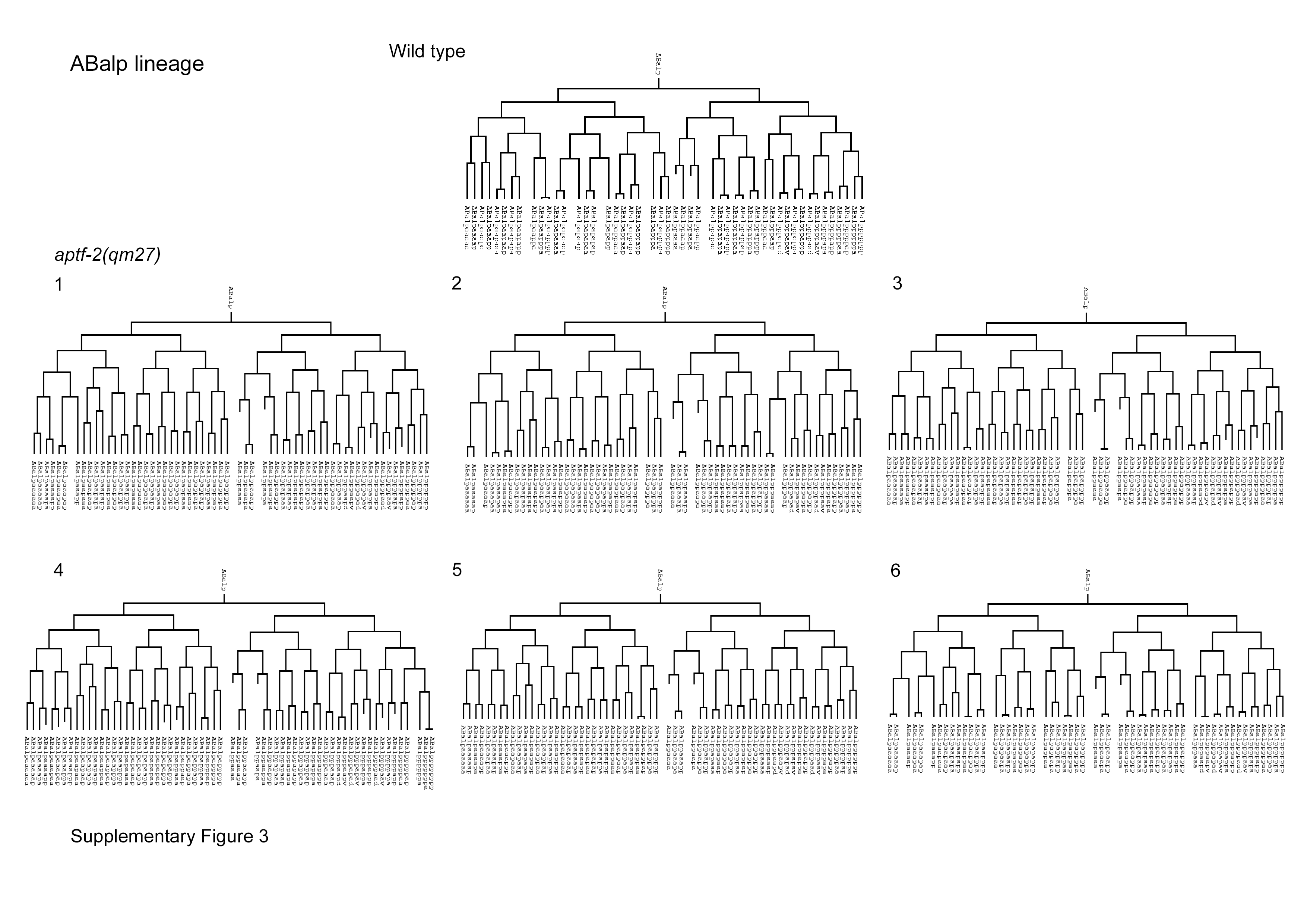

Supplement: S3 Fig — aptf-2(qm27) embryos 1–3 are lineaged to 315 minutes, aptf-2(qm27) embryo 4 to 295 minutes, wild type, aptf-2(qm27) 5 and 6 are lineaged to 270 minutes respectively. (TIF) [file pgen.1006048.s003.tif]

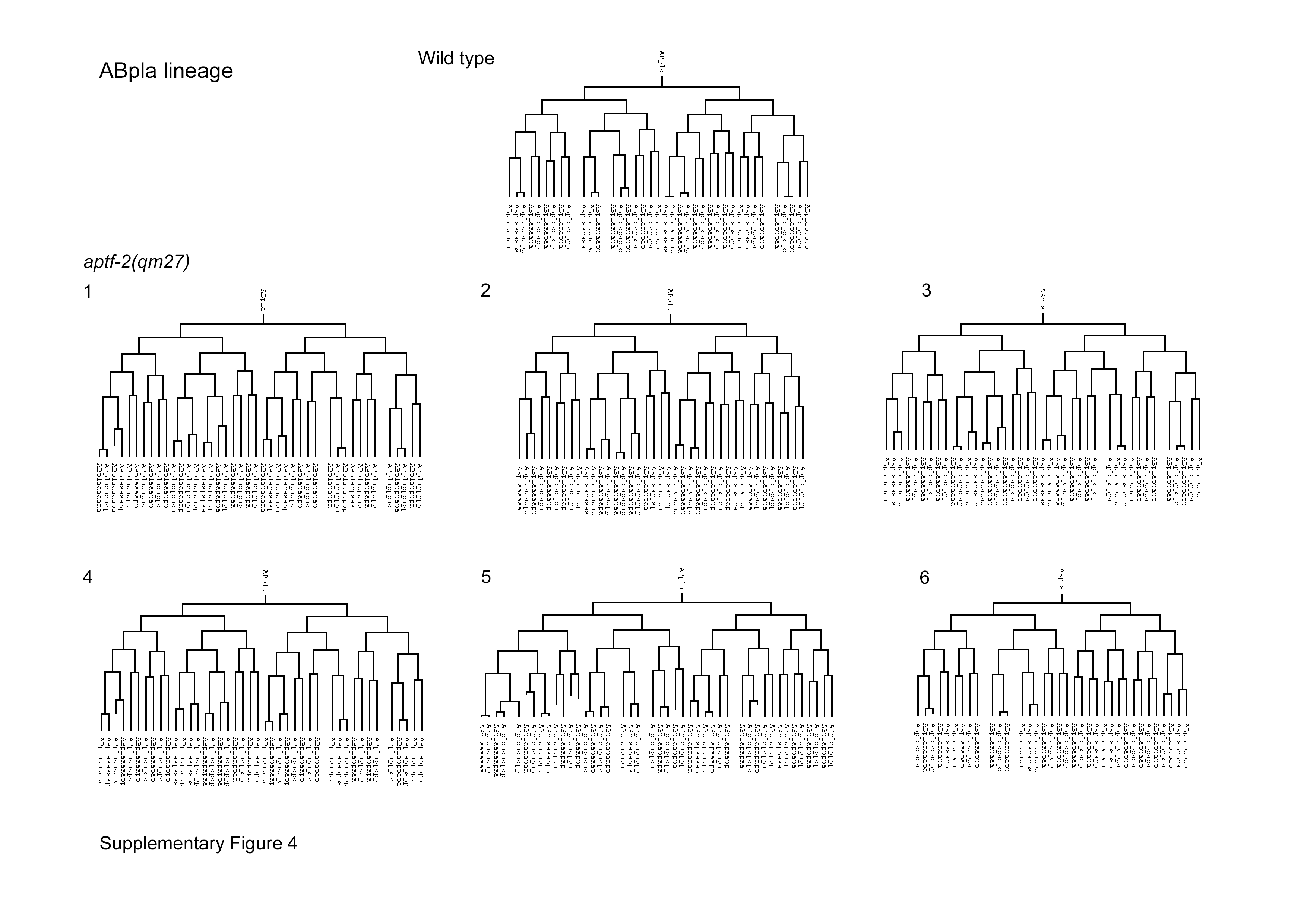

Supplement: S4 Fig — aptf-2(qm27) embryos 1–3 are lineaged to 315 minutes, aptf-2(qm27) embryo 4 to 295 minutes, wild type, aptf-2(qm27) 5 and 6 are lineaged to 270 minutes respectively. (TIF) [file pgen.1006048.s004.tif]

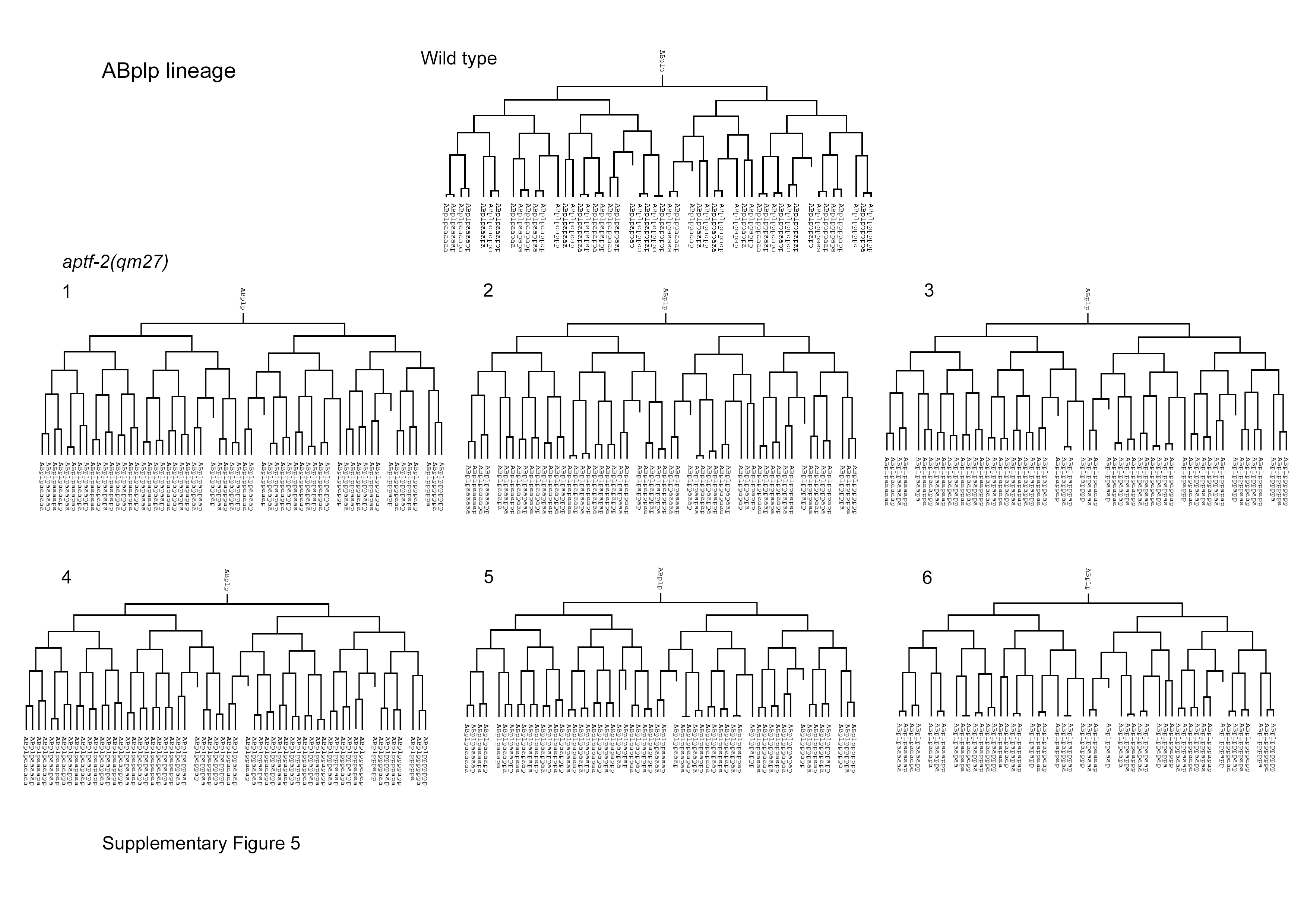

Supplement: S5 Fig — aptf-2(qm27) embryos 1–3 are lineaged to 315 minutes, aptf-2(qm27) embryo 4 to 295 minutes, wild type, aptf-2(qm27) 5 and 6 are lineaged to 270 minutes respectively. (TIF) [file pgen.1006048.s005.tif]

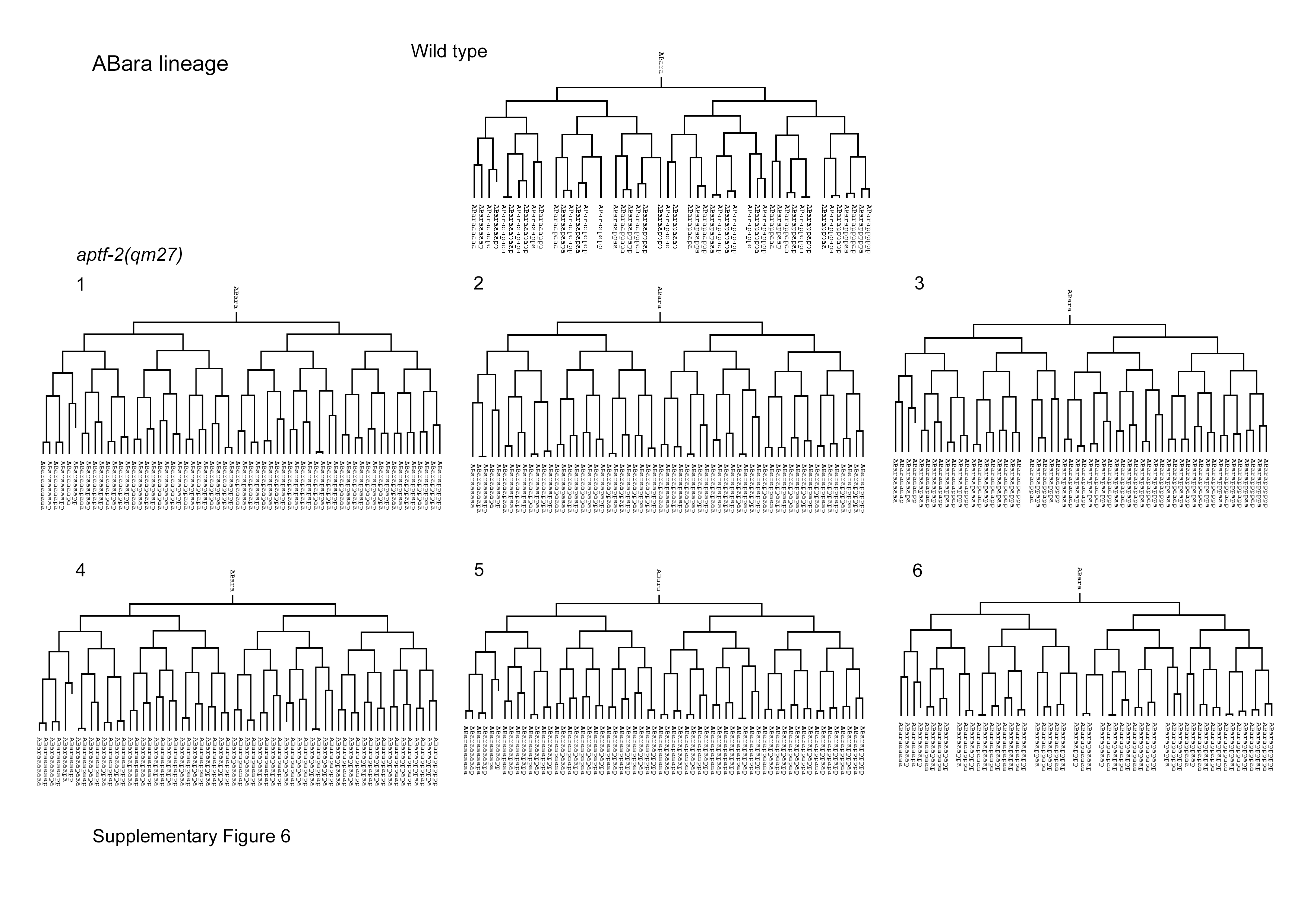

Supplement: S6 Fig — aptf-2(qm27) embryos 1–3 are lineaged to 315 minutes, aptf-2(qm27) embryo 4 to 295 minutes, wild type, aptf-2(qm27) 5 and 6 are lineaged to 270 minutes respectively. (TIF) [file pgen.1006048.s006.tif]

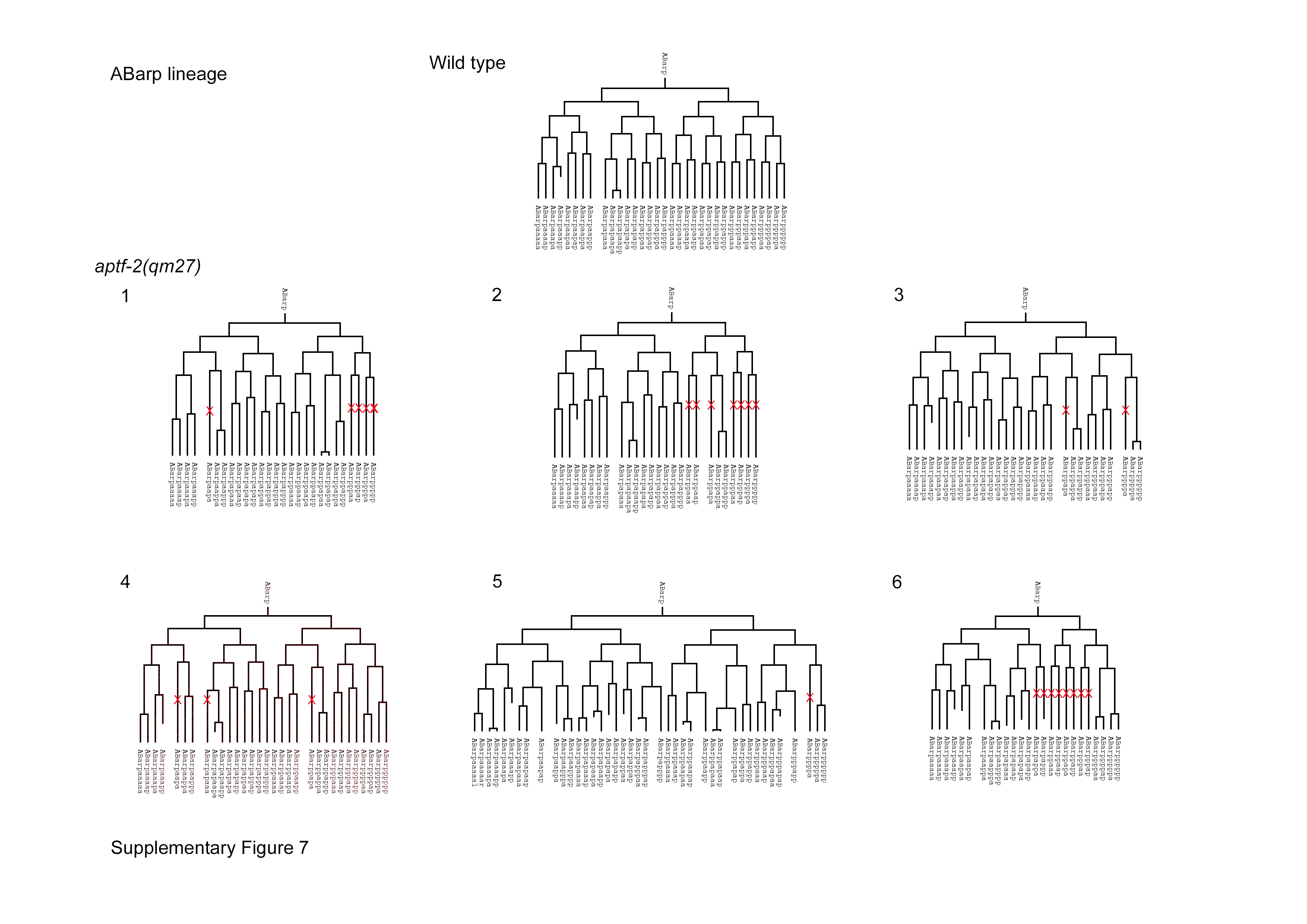

Supplement: S7 Fig — aptf-2(qm27) embryos 1–3 are lineaged to 315 minutes, aptf-2(qm27) embryo 4 to 295 minutes, wild type, aptf-2(qm27) 5 and 6 are lineaged to 270 minutes respectively. Defects in cell division are marked with an X. (TIF) [file pgen.1006048.s007.tif]

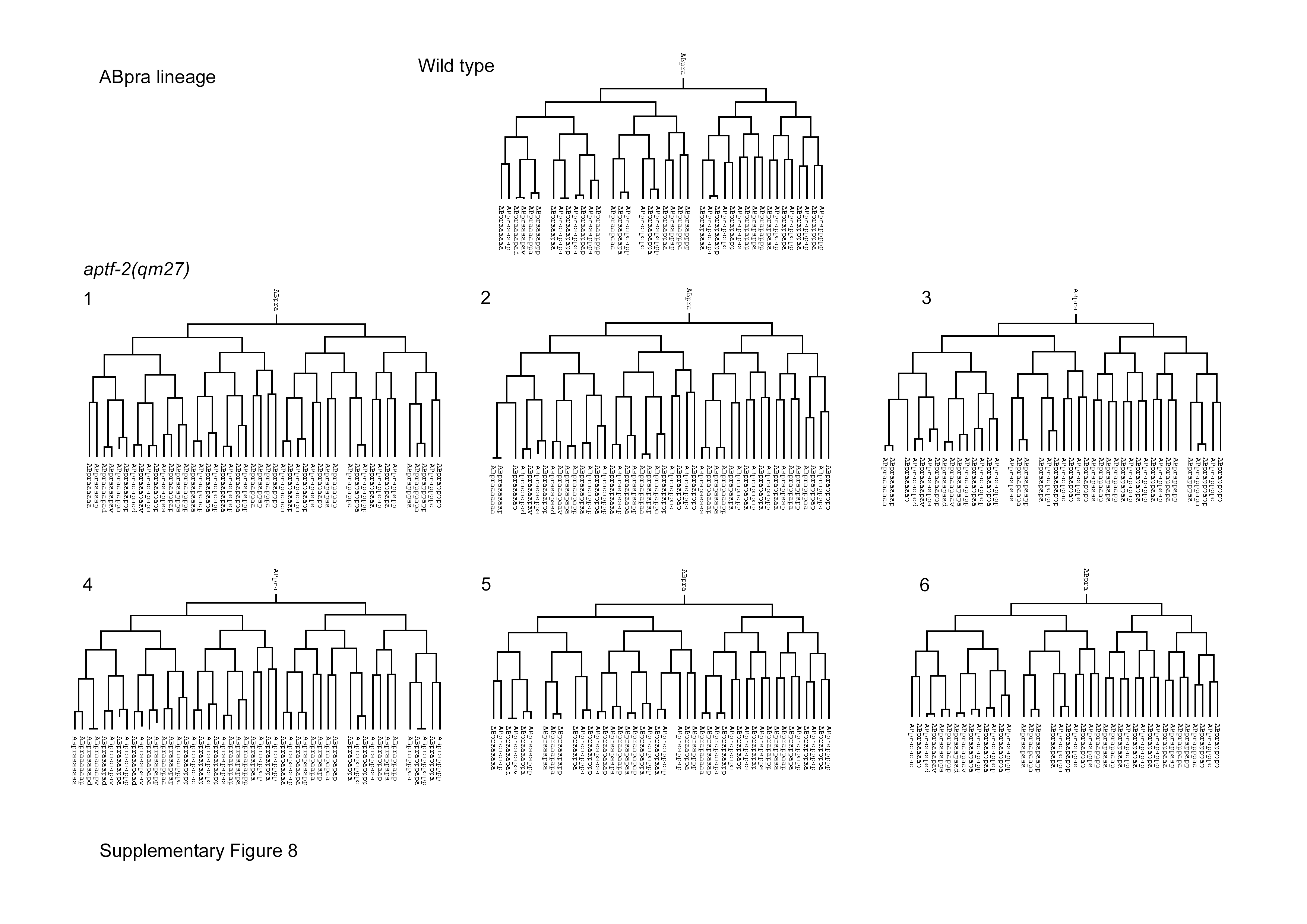

Supplement: S8 Fig — aptf-2(qm27) embryos 1–3 are lineaged to 315 minutes, aptf-2(qm27) embryo 4 to 295 minutes, wild type, aptf-2(qm27) 5 and 6 are lineaged to 270 minutes respectively. (TIF) [file pgen.1006048.s008.tif]

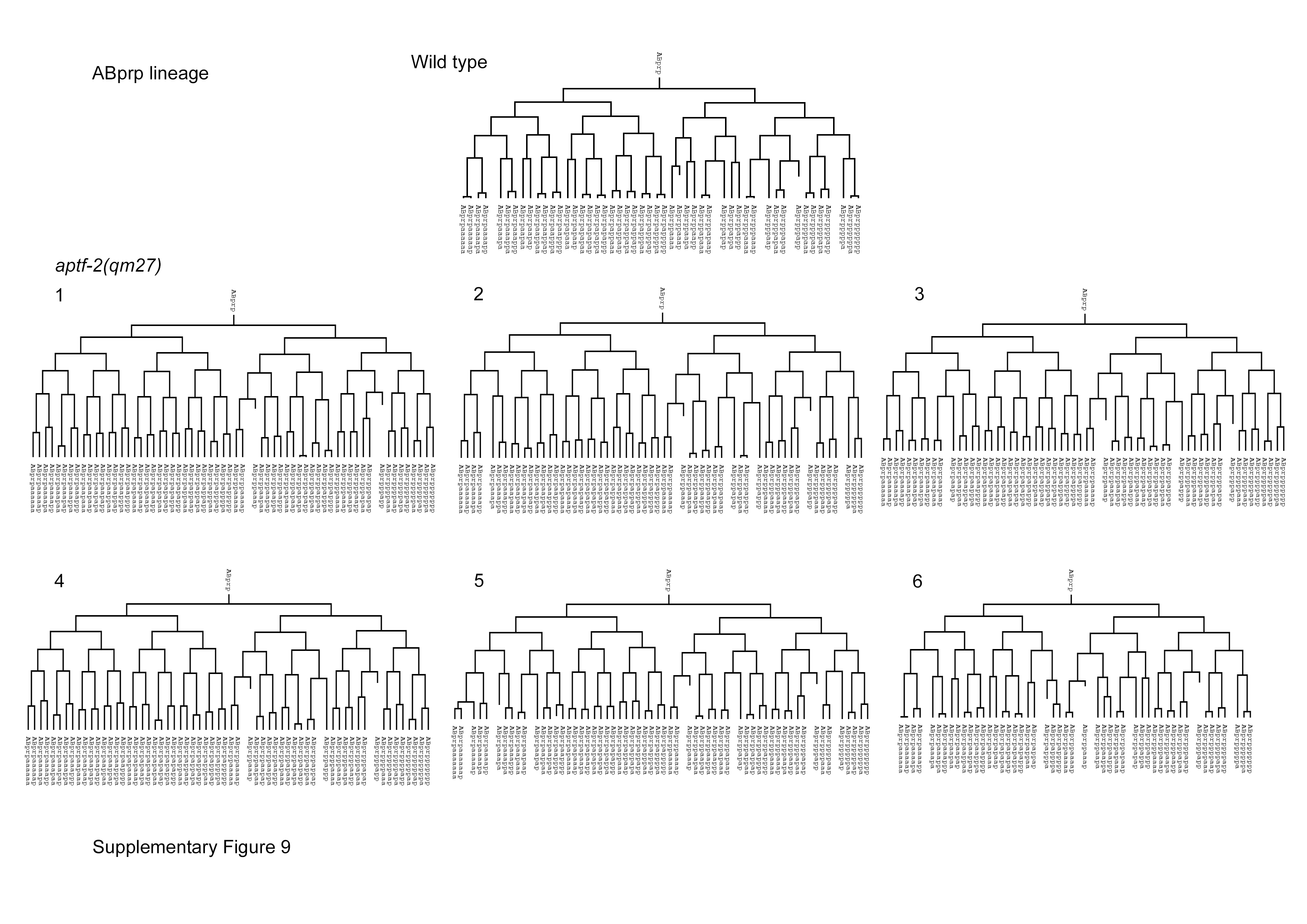

Supplement: S9 Fig — aptf-2(qm27) embryos 1–3 are lineaged to 315 minutes, aptf-2(qm27) embryo 4 to 295 minutes, wild type, aptf-2(qm27) 5 and 6 are lineaged to 270 minutes respectively. (TIF) [file pgen.1006048.s009.tif]

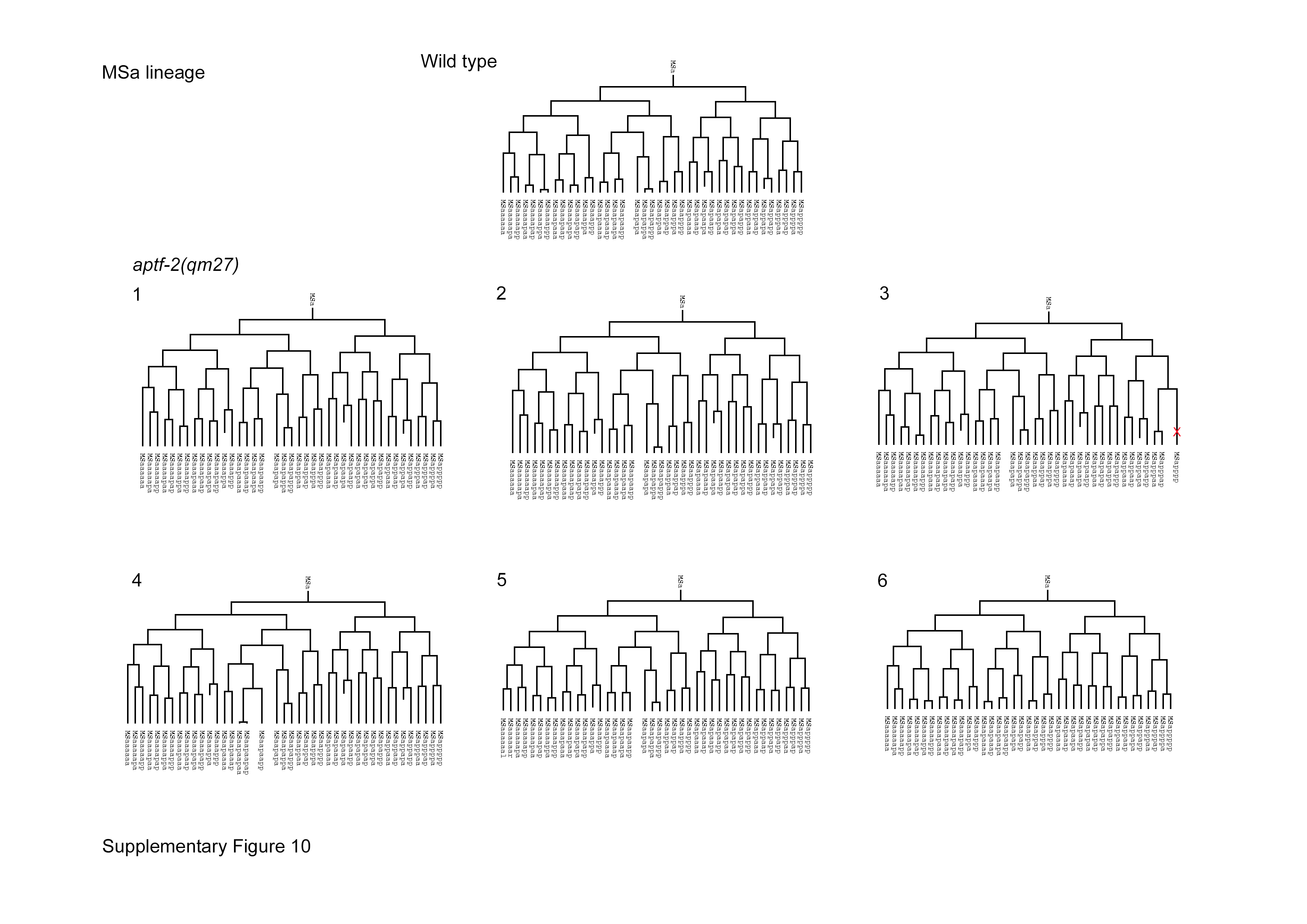

Supplement: S10 Fig — aptf-2(qm27) embryos 1–3 are lineaged to 315 minutes, aptf-2(qm27) embryo 4 to 295 minutes, wild type, aptf-2(qm27) 5 and 6 are lineaged to 270 minutes respectively. (TIF) [file pgen.1006048.s010.tif]

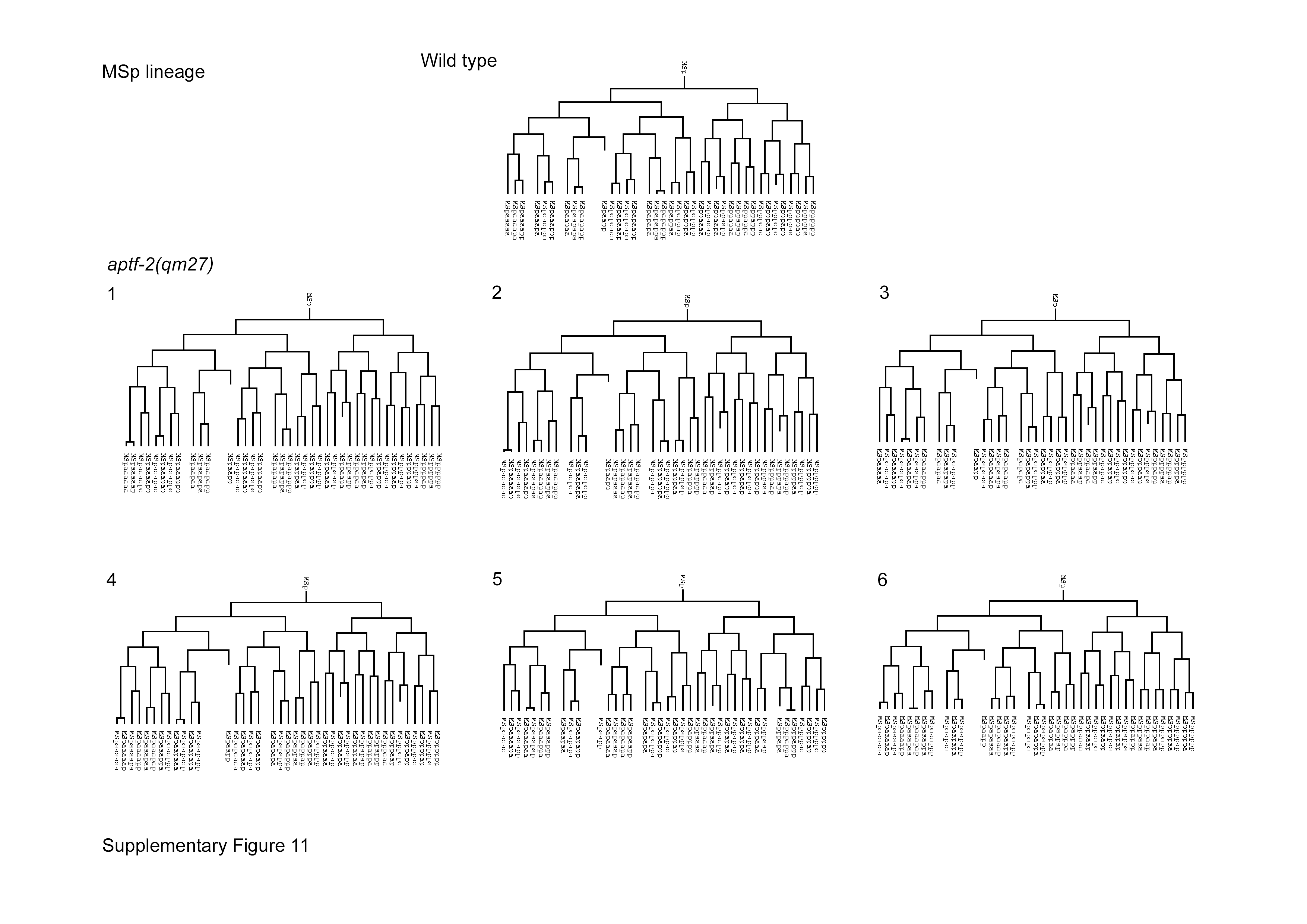

Supplement: S11 Fig — aptf-2(qm27) embryos 1–3 are lineaged to 315 minutes, aptf-2(qm27) embryo 4 to 295 minutes, wild type, aptf-2(qm27) 5 and 6 are lineaged to 270 minutes respectively. (TIF) [file pgen.1006048.s011.tif]

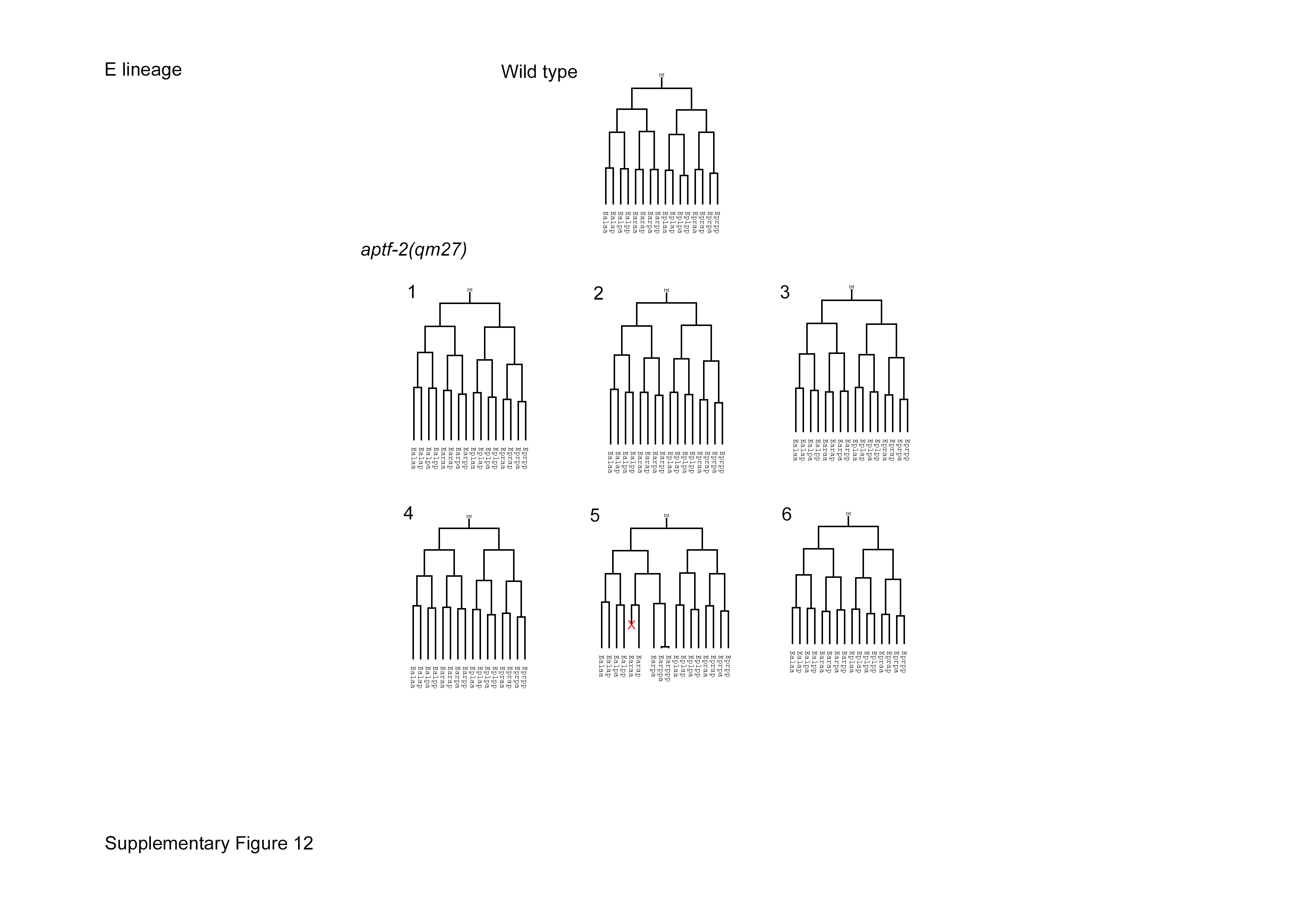

Supplement: S12 Fig — aptf-2(qm27) embryos 1–3 are lineaged to 315 minutes, aptf-2(qm27) embryo 4 to 295 minutes, wild type, aptf-2(qm27) 5 and 6 are lineaged to 270 minutes respectively. Defect in cell division is marked with an X. (TIF) [file pgen.1006048.s012.tif]

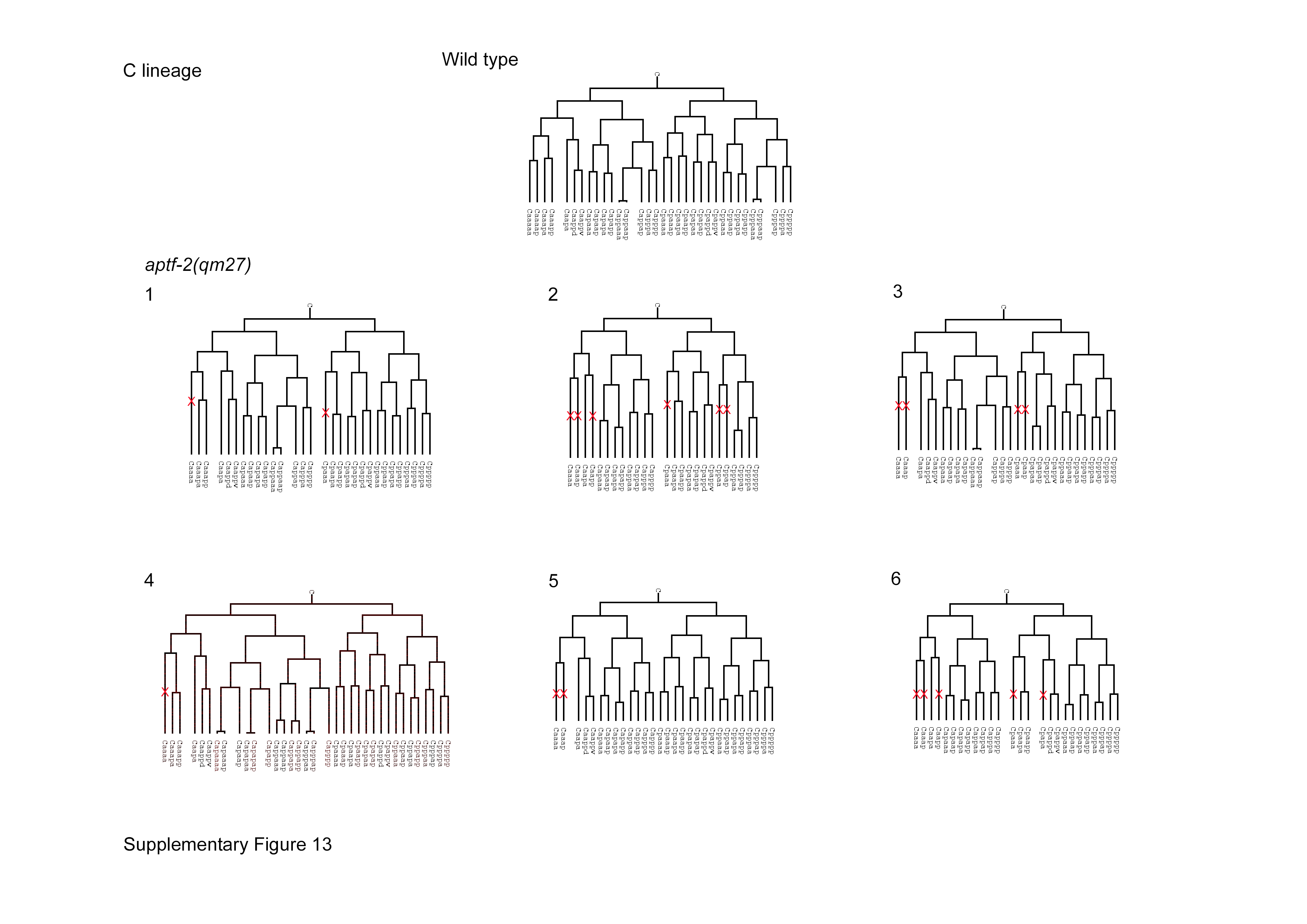

Supplement: S13 Fig — aptf-2(qm27) embryos 1–3 are lineaged to 315 minutes, aptf-2(qm27) embryo 4 to 295 minutes, wild type, aptf-2(qm27) 5 and 6 are lineaged to 270 minutes respectively. Defects in cell division are marked with an X. (TIF) [file pgen.1006048.s013.tif]

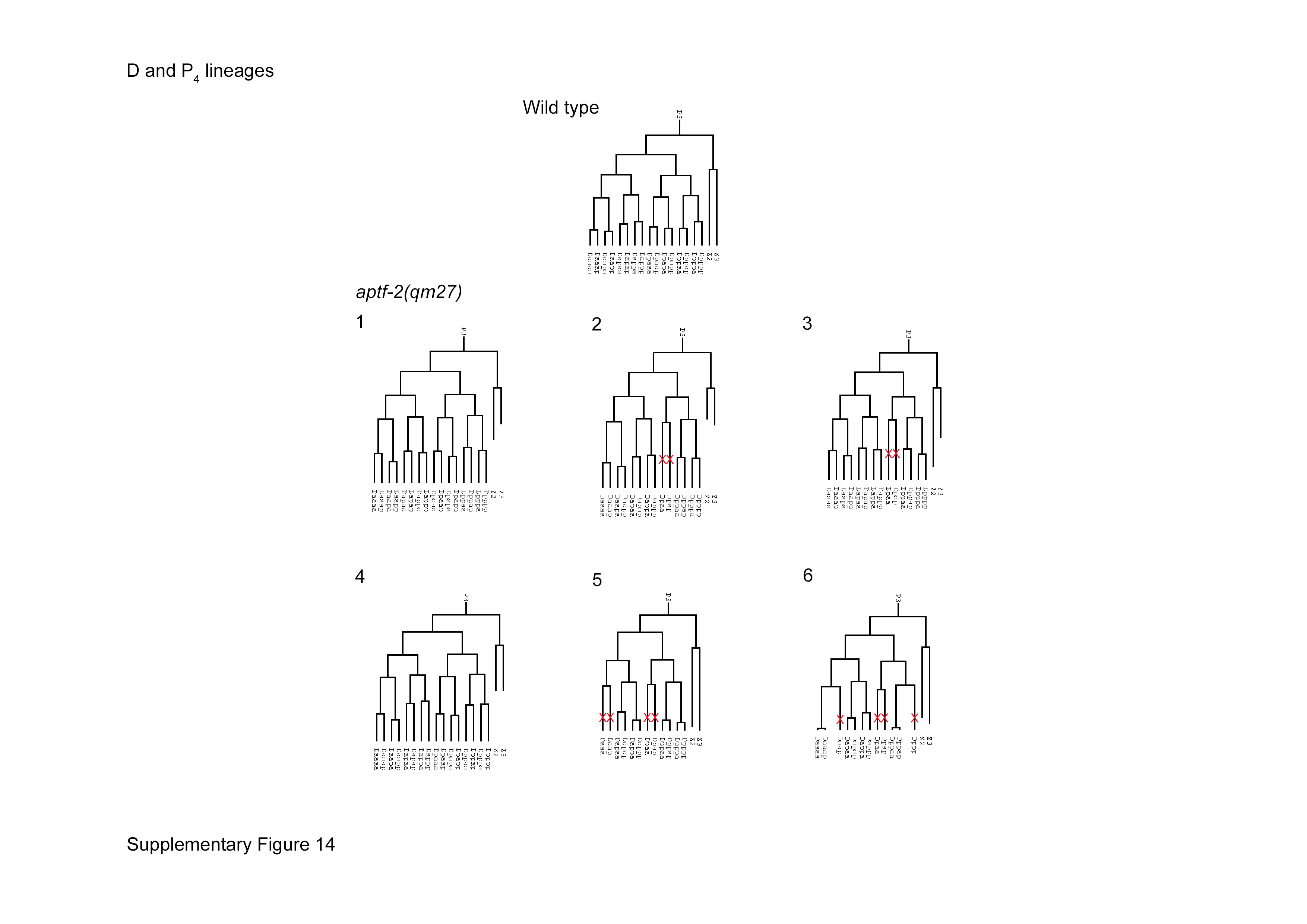

Supplement: S14 Fig — aptf-2(qm27) embryos 1–3 are lineaged to 315 minutes, aptf-2(qm27) embryo 4 to 295 minutes, wild type, aptf-2(qm27) 5 and 6 are lineaged to 270 minutes respectively. Defects in cell division are marked with an X. (TIF) [file pgen.1006048.s014.tif]

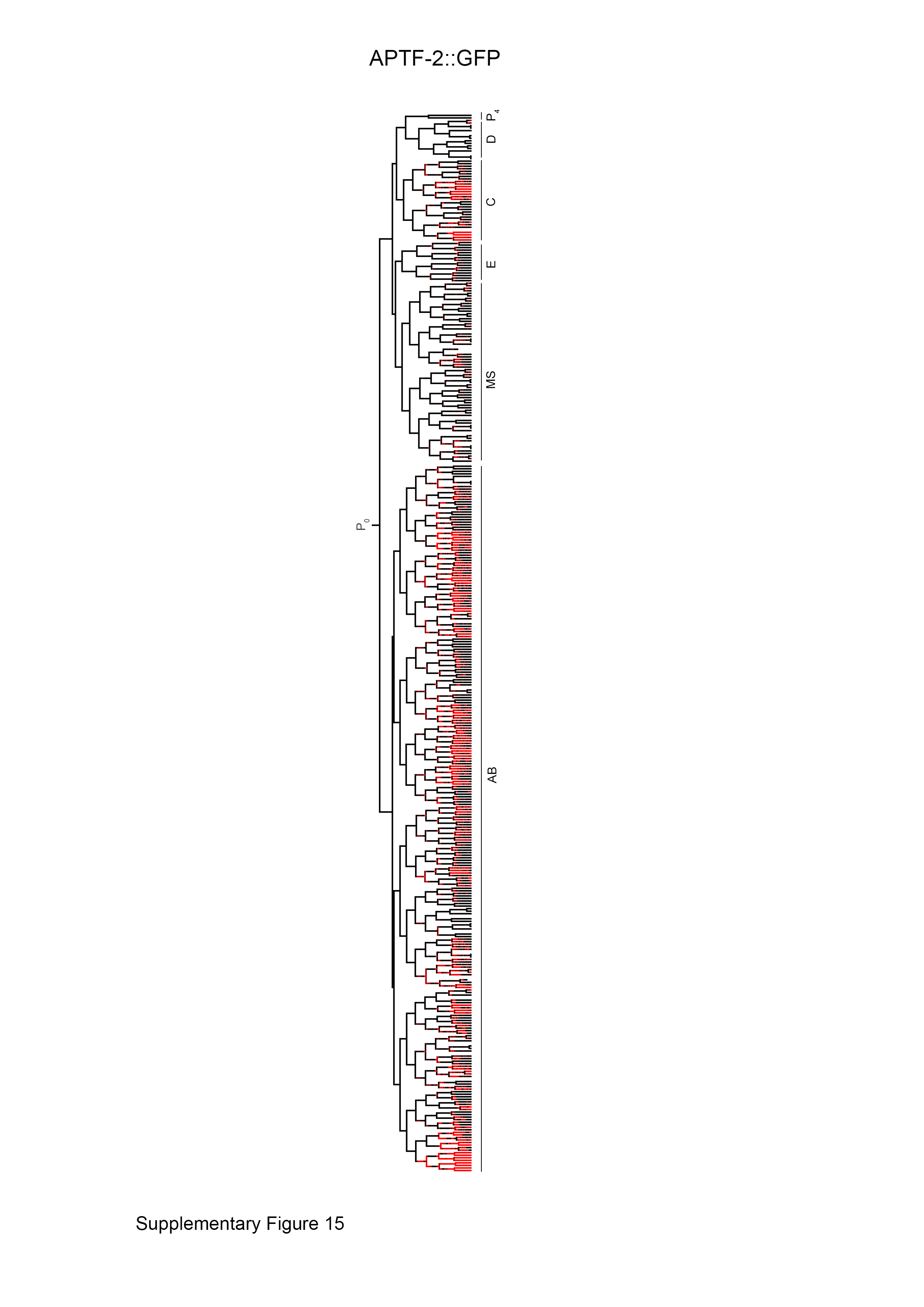

Supplement: S15 Fig — The lineage of an embryo expressing APTF-2::GFP and HIS::mCherry was analyzed for nuclear enrichment of APTF-2::GFP. Nuclear enrichment is represented in red. APTF-2 is enriched in the AB and C lineages during embryogenesis at the time of ventral cleft closure and pre dorsal intercalation. The lineage was analysed to 232 minutes. (TIF) [file pgen.1006048.s015.tif]

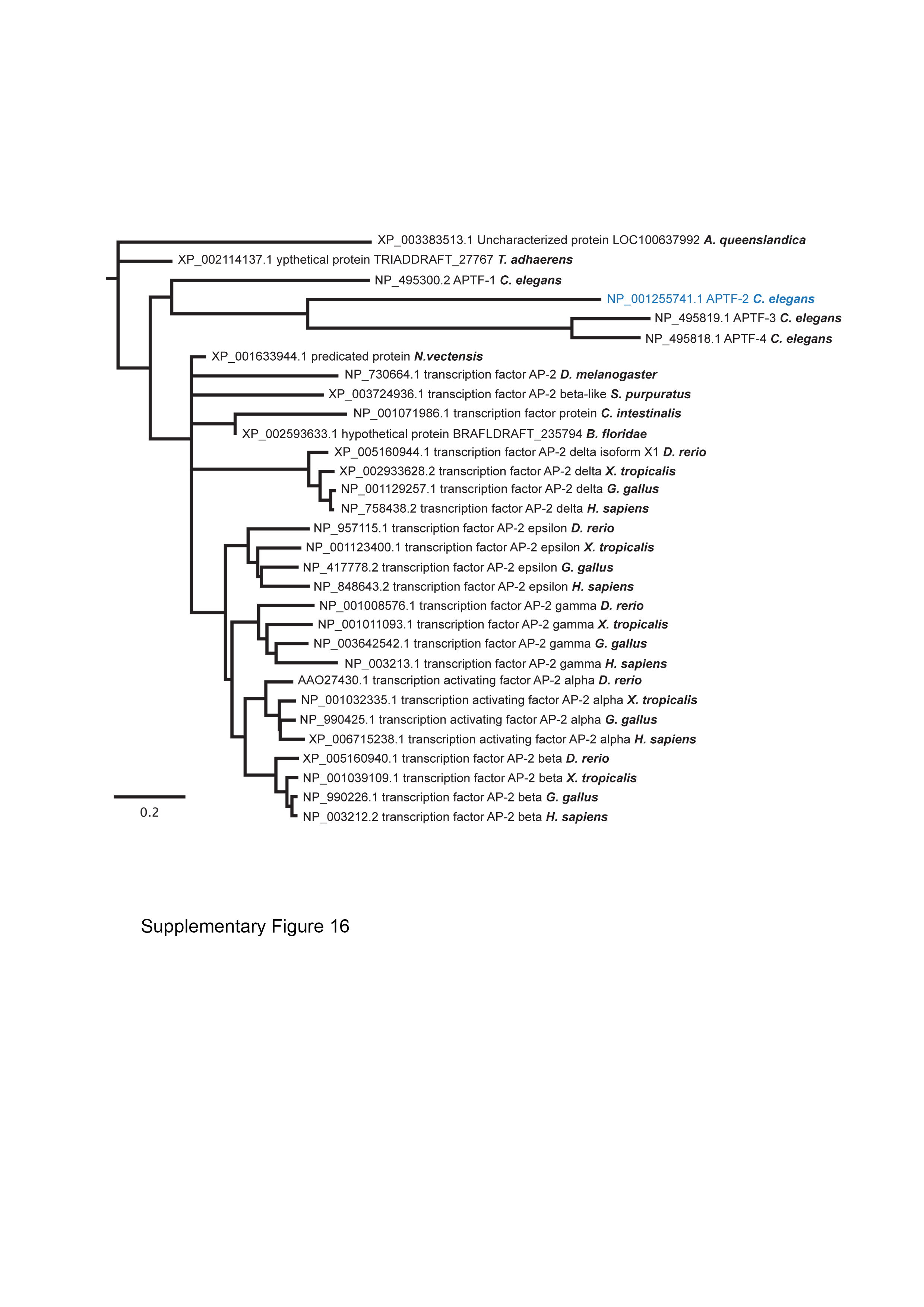

Supplement: S16 Fig — The amino acid sequences were aligned using Constraint-based Multiple Protein Alignment Tool (COBALT) and the phylogenetic tree was built using neighbor-joining method and visualized using Geneious. Species: C. elegans (soil worm), A. queenslandica (sponge), T. adhaerens (a simple metazoan), N. vectensis (sea anemone), B. floridae (lancelet), D. melanogaster (fruit fly), C. intestinalis (tunicate), S. purpuratus (sea urchin), D. rerio (fish), G. gallus (chicken), X. tropicalis (frog), H. sapiens (human). C. elegans APTF-2, which is the subject of this study is highlighted in blue. (TIF) [file pgen.1006048.s016.tif]

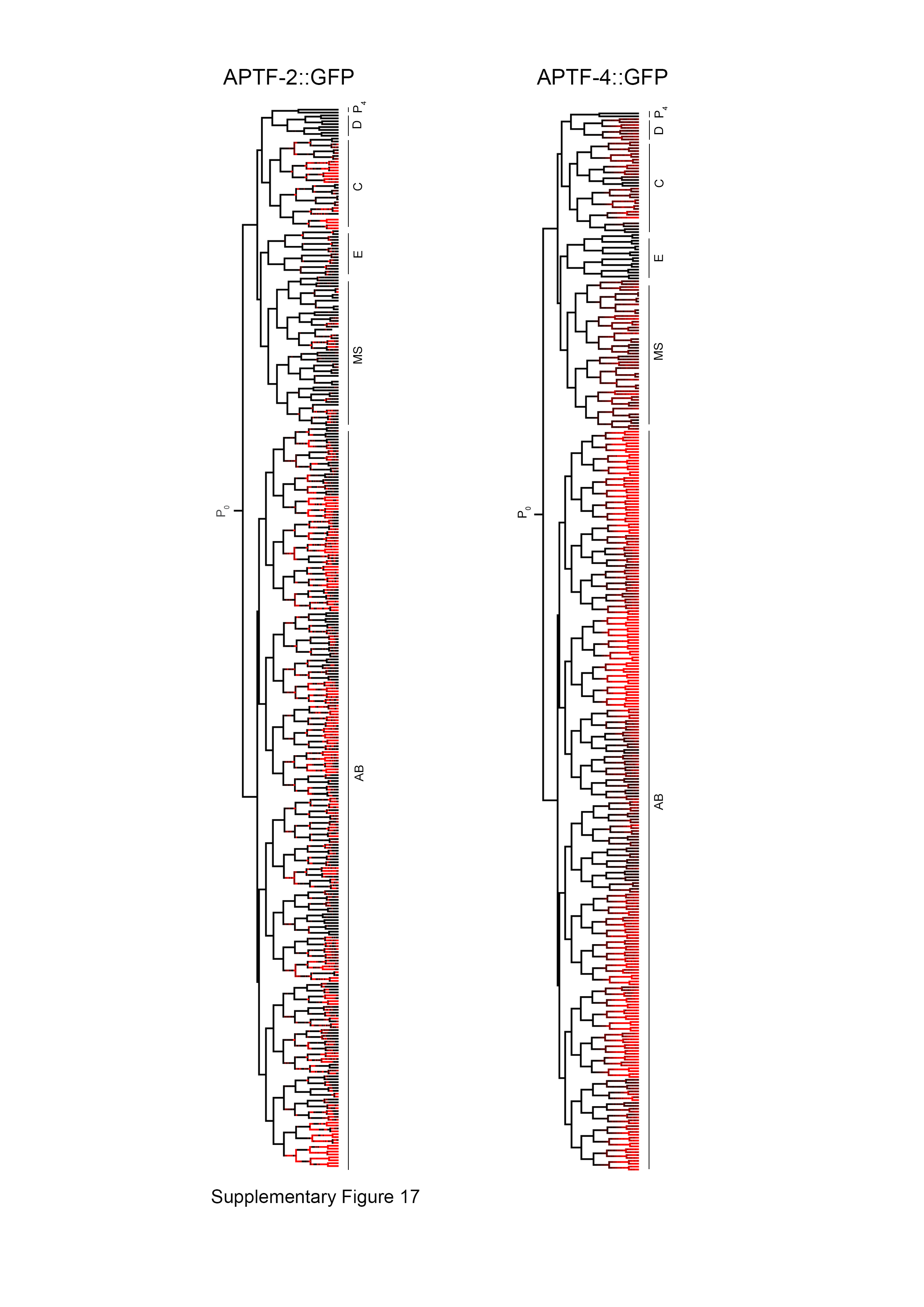

Supplement: S17 Fig — Both lineage trees shows enriched expression in the AB and C lineages while the MS, E and D has weak expression. Trees were drawn to 210 minutes. (TIF) [file pgen.1006048.s017.tif]
