# Supplementary material for: The AP-2 Transcription Factor APTF-2 Is Required for Neuroblast and Epidermal Morphogenesis in Caenorhabditis elegans Embryogenesis
Source: PLoS Genet. 2016 May 13;12(5):e1006048. doi: 10.1371/journal.pgen.1006048 (PMC4866721; doi:10.1371/journal.pgen.1006048)
Supplement: S1 Table — (DOCX) [file pgen.1006048.s018.docx]

**S1 Table. Morphological defects of *aptf-2* mutant worms.**

| Genotype | n | % Larval/ adult worms having the following phenotype: | | | | | |
| --- | --- | --- | --- | --- | --- | --- | --- |
|  |  | Wild- type | Head defect | Tail defect | Head & tail defect | Short body | Middle body defect |
| *aptf-2*(*gk902*) | 19 | 0 | 5 | 5 | 90 | 0 | 0 |
| *aptf-2*(*qm27*) | 597 | 18 | 27 | 28 | 22 | 2 | 3 |

Head defects include lumpy and twisted heads; tail defects include shortened and lumpy tails; middle body defect is defined as a defect occurring anywhere along the body besides the head and tail.
