# Supplementary material for: The AP-2 Transcription Factor APTF-2 Is Required for Neuroblast and Epidermal Morphogenesis in Caenorhabditis elegans Embryogenesis
Source: PLoS Genet. 2016 May 13;12(5):e1006048. doi: 10.1371/journal.pgen.1006048 (PMC4866721; doi:10.1371/journal.pgen.1006048)
Supplement: S2 Table — (DOCX) [file pgen.1006048.s019.docx]

**S2 Table. Expression of APTF-2::GFP in *aptf-2*(*gk902*) and *aptf-2*(*qm27*) animals rescues their embryonic lethality.**

| Genotypes  n ≥ 500 embryos (≥ 60 animals) | % Embryonic lethality | P values wild-type | P values *aptf-2*(*qm27*) | P values *aptf-2*(*gk902*) |
| --- | --- | --- | --- | --- |
| Wild-type | 0.3 ± 0.4 | - | - | - |
| *aptf-2*(*gk902*) | 99 ± 0.5 | 5.2 x 10^-12^ | - | - |
| *aptf-2*(*qm27*) | 56 ± 7 | 7.0 x 10^-33^ | - | - |
| *aptf-2*(*gk902*)*/aptf-2*(*qm27*) | 68 ± 2 | - | - | - |
| *In[aptf-2p::aptf-2::gfp]*; *aptf-2*(*gk902*) | 2.7 ± 2.8 | - | - | 1.4 x 10^-17^ |
| *In[aptf-2p::aptf-2::gfp]*; *aptf-2*(*qm27*) | 0.3 ± 0.6 | - | 5.3 x 10^-7^ | - |

Mean% embryonic lethality ± s.e.m. is indicated. The two-tailed Student’s *t*-test was applied to compare % embryonic lethality of *aptf-2*mutants to that of wild-type and % embryonic lethality of *aptf-2* mutants expressing APTF-2::GFP to that of the corresponding *aptf-2* mutants. Values from wild-type, *aptf-2(gk902)* and *aptf-2(qm27)* are replicated from Table 1.
