# Supplementary material for: The AP-2 Transcription Factor APTF-2 Is Required for Neuroblast and Epidermal Morphogenesis in Caenorhabditis elegans Embryogenesis
Source: PLoS Genet. 2016 May 13;12(5):e1006048. doi: 10.1371/journal.pgen.1006048 (PMC4866721; doi:10.1371/journal.pgen.1006048)
Supplement: S3 Table — (DOCX) [file pgen.1006048.s020.docx]

**S3A Table. APTF-2 is required for timely embryogenesis events (dorsally-oriented embryos).**

| Genotypes  n ≥ 10 embryos | Embryogenesis events (minutes) | | |
| --- | --- | --- | --- |
|  | Ea/Ep ingression | Dorsal intercalation | Elongation 2 folds |
| Wild-type | 54 ± 2.0 | 213 ± 4.9 | 92 ± 4.7 |
| *aptf-2*(*qm27*) | 59 ± 3.9 | 246 ± 9.2 | 148 ± 16 |
| *In[aptf-2p::aptf-2::gfp]*; *aptf-2*(*qm27*) | 59 ± 3.3 | 232 ± 5.1 | 95 ± 5.4 |

**S3B Table. APTF-2 is required for timely embryogenesis events (ventrally-oriented embryos).**

| Genotypes  n ≥ 10 embryos | Embryogenesis events (minutes) | | | | |
| --- | --- | --- | --- | --- | --- |
|  | Ea/Ep ingression | Ventral cleft starts | Ventral cleft closes | Ventral enclosure | Elongation 2 folds |
| Wild-type | 54 ± 2.6 | 111 ± 3.7 | 20 ± 3.3 | 107 ± 4.8 | 64 ± 7.4 |
| *aptf-2*(*qm27*) | 59 ± 2.7 | 133 ± 3.7 | 60 ± 11 | 108 ± 14 | 98 ± 16 |
| *In[aptf-2p::aptf-2::gfp]*; *aptf-2*(*qm27*) | 60 ± 2.5 | 111 ± 4.0 | 30 ± 5.5 | 123 ± 7.3 | 73 ± 3.4 |

The timing of each embryogenesis event is measured by subtracting the real timing of a given event with the real timing of the preceding event.
