# Supplementary material for: The AP-2 Transcription Factor APTF-2 Is Required for Neuroblast and Epidermal Morphogenesis in Caenorhabditis elegans Embryogenesis
Source: PLoS Genet. 2016 May 13;12(5):e1006048. doi: 10.1371/journal.pgen.1006048 (PMC4866721; doi:10.1371/journal.pgen.1006048)
Supplement: S4 Table — (DOCX) [file pgen.1006048.s021.docx]

**S4 Table. Affected lineages in *aptf-2*(*qm27*) mutant embryos .**

| Lineage | Cell | Fate |
| --- | --- | --- |
| ABarp | ABarpaaaaa | QLQsoDL and QLQsoDR ring gang |
|  | ABarpaaaap | QLQshDL and CEPshDL ring gang |
|  | ABarpaaapa | QLQshDR and CEPshDR ring gang |
|  | ABarpaaapp | Death |
|  | *ABarpaapaa* | *hyp 6 epidermal cell* |
|  | *ABarpaapap* | *hyp 7 epidermal cell* |
|  | ABarpaappa | hyp 7 epidermal cell |
|  | ABarpaappp | hyp 7 epidermal cell |
|  | ABarpapaaaa | URADR and CEMDR ring gang |
|  | ABarpapaap | Death, CEPDR and URXR ring gang |
|  | ABarpapapa | hyp 4 epidermal cell |
|  | ABarpapapp | hyp 6 epidermal cell |
|  | *ABarpappaa* | *XXXR hyp* |
|  | *ABarpappap* | *hyp 5 epidermal cell* |
|  | *ABarpapppa* | *H0R seam epidermal cell* |
|  | *ABarpapppp* | *H1R seam epidermal cell* |
|  | *ABarppaaaa* | *ADEshL ring gang* |
|  | *ABarppaaap* | *H2L seam epidermal cell* |
|  | *ABarppaapa* | *hyp 7 epidermal cell* |
|  | *ABarppaapp* | *BDUL and ALML lateral neuron* |
|  | *ABarppapaa* | *V1L seam epidermal cell* |
|  | *ABarppapap* | *V2L seam epidermal cell* |
|  | *ABarppappa* | *V4L seam epidermal cell* |
|  | *ABarppappp* | *V6L seam epidermal cell* |
|  | *ABarpppaaa* | *ADEshR ring gang* |
|  | *ABarpppaap* | *H2R seam epidermal cell* |
|  | *ABarpppapa* | *hyp 7 epidermal cell* |
|  | *ABarpppapp* | *BDUR and ALMR lateral neuron* |
|  | *ABarppppaa* | *V1R seam epidermal cell* |
|  | *ABarppppap* | *V2R seam epidermal cell* |
|  | *ABarpppppa* | *V4R seam epidermal cell* |
|  | *ABarpppppp* | *V6R seam epidermal cell* |
| C | *Caaaaa* | *hyp 7 epidermal cell* |
|  | *Caaaap* | *hyp 7 epidermal cell* |
|  | *Caaapa* | *hyp 7 epidermal cell* |
|  | *Caaapp* | *hyp 7 epidermal cell* |
|  | Caapa | Death, DVC d-r gang |
|  | *Caappd* | *hyp 7 epidermal cell* |
|  | *Caappv* | *PVR lumb gang* |
|  | Capaaa | Body muscle, body muscle |
|  | Capaap | Body muscle, body muscle |
|  | Capapa | Body muscle, body muscle |
|  | Capapp | Body muscle, body muscle |
|  | Cappaa | Body muscle, body muscle |
|  | Cappap | Body muscle, body muscle |
|  | Capppa | Body muscle, body muscle |
|  | Capppp | Body muscle, body muscle |
|  | *Cpaaaa* | *hyp 7 epidermal cell* |
|  | *Cpaaap* | *hyp 7 epidermal cell* |
|  | *Cpaapa* | *hyp 7 epidermal cell* |
|  | *Cpaapp* | *hyp 7 epidermal cell* |
|  | *Cpapaa* | *hyp 7 epidermal cell* |
|  | *Cpapap* | *hyp 7 epidermal cell* |
|  | Cpappd | hyp 7 epidermal cell |
|  | Cpappv | hyp 11 epidermal cell |
|  | *Cppaaa* | *Body muscle, body muscle* |
|  | *Cppaap* | *Body muscle, body muscle* |
|  | *Cppapa* | *Body muscle, body muscle* |
|  | *Cppapp* | *Body muscle, body muscle* |
|  | Cpppaa | Body muscle, body muscle |
|  | Cpppap | Body muscle, body muscle |
|  | Cppppa | Body muscle, body muscle |
|  | Cppppp | Body muscle, body muscle |
| D | Daaaa | Embryonic body wall muscle |
|  | Daaap | Embryonic body wall muscle |
|  | Daapa | Embryonic body wall muscle |
|  | Daapp | Embryonic body wall muscle |
|  | Dapaa | Embryonic body wall muscle |
|  | Dapap | Embryonic body wall muscle |
|  | Dappa | Embryonic body wall muscle |
|  | Dappp | Embryonic body wall muscle |
|  | Dpaaa | Embryonic body wall muscle |
|  | Dpaap | Embryonic body wall muscle |
|  | Dpapa | Embryonic body wall muscle |
|  | Dpapp | Embryonic body wall muscle |
|  | Dppaa | Embryonic body wall muscle |
|  | Dppap | Embryonic body wall muscle |
|  | Dppppa | Embryonic body wall muscle |
|  | Dppppp | Embryonic body wall muscle |

Cells that are affected in *aptf-2*(*qm27*) embryos and their fates are shown in italics and highlighted in color according to the same color scheme shown in figure 6, reflecting how many of the analysed embryos showed this defect.
