# Supplementary material for: The AP-2 Transcription Factor APTF-2 Is Required for Neuroblast and Epidermal Morphogenesis in Caenorhabditis elegans Embryogenesis
Source: PLoS Genet. 2016 May 13;12(5):e1006048. doi: 10.1371/journal.pgen.1006048 (PMC4866721; doi:10.1371/journal.pgen.1006048)
Supplement: S5 Table — (DOCX) [file pgen.1006048.s022.docx]

**S5 Table. Nuclear targeting of APTF-2 is sufficient to rescue *aptf-2*(*gk902*) and *aptf-2*(*qm27*) embryonic lethality.**

| Genotypes  n ≥ 500 embryos (≥ 60 animals) | % Embryonic lethality | P values  Wild-type | P values *aptf-2*(*qm27*) | P values *aptf-2*(*gk902*) |
| --- | --- | --- | --- | --- |
| Wild-type | 0.3 ± 0.4 | - | - | - |
| *aptf-2*(*gk902*) | 99 ± 0.5 | 5.2 x 10^-12^ | - | - |
| *aptf-2*(*qm27*) | 56 ± 7 | 7.0 x 10^-33^ | - | - |
| *Ex[aptf-2p::aptf-2::gfp]*; *aptf-2*(*qm27*) | 36 ± 14 | - | 1.4 x 10^-2^ | - |
| *Ex[aptf-2p::aptf-2::NLS::gfp]*; *aptf-2*(*gk902*) | 51 ± 11 | - | - | 1.2 x 10^-7^ |
| *Ex[aptf-2p::aptf-2::NLS::gfp]*; *aptf-2*(*qm27*) | 17 ± 4.6 | - | 1.6 x 10^-6^ | - |

Mean% embryonic lethality ± s.e.m. is indicated. The two-tailed Student’s *t*-test was applied to compare % embryonic lethality of *aptf-2* mutants to that of wild-type and % embryonic lethalityof *aptf-2* mutants expressing APTF-2::NLS::GFP to that of the corresponding *aptf-2* mutants.
