# Supplementary material for: The AP-2 Transcription Factor APTF-2 Is Required for Neuroblast and Epidermal Morphogenesis in Caenorhabditis elegans Embryogenesis
Source: PLoS Genet. 2016 May 13;12(5):e1006048. doi: 10.1371/journal.pgen.1006048 (PMC4866721; doi:10.1371/journal.pgen.1006048)
Supplement: S6 Table — (DOCX) [file pgen.1006048.s023.docx]

**S6 Table. APTF-2 and APTF-4 synergistically regulate *C. elegans* embryogenesis**

| Genotypes  n ≥ 500 embryos (≥ 60 animals) | % Embryonic lethality | P values wild-type | P values *aptf-2*(*qm27*) |
| --- | --- | --- | --- |
| Wild-type | 0.3 ± 0.4 | - | - |
| *aptf-2*(*qm27*) | 56 ± 7 | 7.0 x 10^-33^ | - |
| *aptf-4*(*RNAi*) | 26 ± 2.9 | 4.2 x 10^-12^ | - |
| *aptf-2*(*qm27*); *aptf-4*(*RNAi*) | 99 ± 0.3 | - | 4.8 x 10^-5^ |

Mean % embryonic lethality ± s.e.m. is indicated. The two-tailed Student’s *t*-test was applied to compare % embryonic lethality of *aptf-2*(*qm27*) mutants and *aptf-3/4*(*RNAi*) to that of wild-type, as well as, % embryonic lethalityof *aptf-2*(*qm27*) treated with *aptf-4*(*RNAi*) to that of *aptf-2*(*qm27*).
