# Supplementary material for: The AP-2 Transcription Factor APTF-2 Is Required for Neuroblast and Epidermal Morphogenesis in Caenorhabditis elegans Embryogenesis
Source: PLoS Genet. 2016 May 13;12(5):e1006048. doi: 10.1371/journal.pgen.1006048 (PMC4866721; doi:10.1371/journal.pgen.1006048)
Supplement: S7 Table — (DOCX) [file pgen.1006048.s024.docx]

**S7 Table. Phenotypic analysis of *aptf-2*(*qm27*) embryos injected with *aptf-4* dsRNA and analyzed by DIC.**

| Embryo orientation | n | % Embryonic phenotypes | | | | |
| --- | --- | --- | --- | --- | --- | --- |
|  |  | Wild-type | Dorsal intercalation defect | Ventral enclosure defect | Elongation arrest | Defective elongation |
| Dorsal | 7 | 0 | 100 | n.a. | 100 | 0 |
| Ventral | 14 | 0 | n.a. | 57 | 100 | 0 |
